# Sterols from the Octocoral *Nephthea columnaris*

**DOI:** 10.3390/md15070212

**Published:** 2017-07-04

**Authors:** Ta-Yuan Whuang, Hong-Chieh Tsai, Yin-Di Su, Tsong-Long Hwang, Ping-Jyun Sung

**Affiliations:** 1 Graduate Institute of Marine Biology, National Dong Hwa University, Pingtung 944, Taiwan; xdanielzero@gmail.com; 2 National Museum of Marine Biology and Aquarium, Pingtung 944, Taiwan; gobetter04@gmail.com; 3 Graduate Institute of Clinical Medical Sciences, College of Medicine, Chang Gung University, Taoyuan 333, Taiwan; Newcomer9999@gmail.com; 4 Department of Neurosurgery, Chang Gung Memorial Hospital, Taoyuan 333, Taiwan; 5 Graduate Institute of Natural Products, College of Medicine and Chinese Herbal Medicine Research Team, Healthy Aging Research Center, Chang Gung University, Taoyuan 333, Taiwan; 6 Research Center for Chinese Herbal Medicine, Research Center for Food and Cosmetic Safety, and Graduate Institute of Health Industry Technology, College of Human Ecology, Chang Gung University of Science and Technology, Taoyuan 333, Taiwan; 7 Department of Anesthesiology, Chang Gung Memorial Hospital, Taoyuan 333, Taiwan; 8 Chinese Medicine Research and Development Center, China Medical University Hospital, Taichung 404, Taiwan; 9 Graduate Institute of Natural Products, Kaohsiung Medical University, Kaohsiung 807, Taiwan; 10 Department of Marine Biotechnology and Resources, National Sun Yat-sen University, Kaohsiung 804, Taiwan

**Keywords:** octocoral, *Nephthea columnaris*, columnaristerol, litosterol, superoxide anion, elastase

## Abstract

Two new sterols, columnaristerols B (**1**) and C (**2**), along with two known analogues, 5,6-epoxylitosterol (**3**) and litosterol (**4**), were obtained from the octocoral *Nephthea columnaris*. The structures of new sterols **1** and **2** were elucidated by using spectroscopic methods and comparing the spectroscopic data with those of known related metabolites. Sterol **3** was found to suppress superoxide anion production and elastase secretion by human neutrophils.

## 1. Introduction

**Figure 1:**
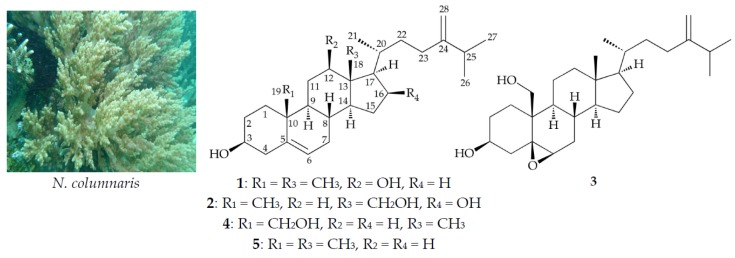
Octocoral *N. columnaris* and structures of columnaristerols B (**1**), C (**2**), 5,6-epoxylitosterol (**3**), litosterol (**4**), and 24-methylenecholesterol (**5**).

The octocoral *Nephthea columnaris* (Studer, 1895) (family Nephtheidae, order Alcyonacea, class Anthozoa, phylum Cnidaria) comprises large quantities of terpenoid [1,2] and steroid [3,4] analogues, which often have complex structures and biological activities. In continuing studies of the constituents of *N. columnaris* collected from the southern waters off the coast of Taiwan, two new sterols, columnaristerols B (**1**) and C (**2**), and known analogues, 5,6-epoxylitosterol (**3**) and litosterol (**4**) [5] (Figure 1), were obtained. Sterol **3** was found to inhibit the production of superoxide anions and release of elastase by human neutrophils.

## 2. Results and Discussion

Columnaristerol B (**1**) was obtained as a non-crystalline powder, and high-resolution electrospray ionization mass spectrum (HRESIMS) analysis revealed that **1** had a pseudomolecular peak at *m/z* 437.33918 (calcd. for C_28_H_46_O_2_ + Na, 437.33900), which established the molecular formula C_28_H_46_O_2_, indicating six degrees of unsaturation. Data from ^1^H NMR and distortionless enhancement of polarization transfer (DEPT) analyses and examination along with the molecular formula of the compound suggested that there were two exchangeable protons that required the presence of two hydroxyl groups. IR spectrum analysis showed a broad absorption at 3386 cm^−1^, which further confirmed that interpretation. Combined examination of the molecular formula, ^13^C NMR and DEPT data revealed the presence of 28 carbons in **1** (Table 1), including five methyls, nine sp^3^ methylenes, eight sp^3^ methines (including two oxymethines, δ_C_ 79.5 and 71.7), an exocyclic double bond (δ_C_ 156.8 and 106.0), a trisubstituted double bond (δ_C_ 140.6 and 121.6), and two sp^3^ quaternary carbons.

In addition, the ^1^H NMR spectrum (Table 1) exhibited five methyl signals at δ_H_ 1.04 (3H, d, *J* = 6.4 Hz), 1.03 (3H, d, *J* = 6.8 Hz), 1.02 (3H, d, *J* = 6.8 Hz), 1.02 (3H, s), and 0.72 (3H, s). As the trisubstituted double bond and exocyclic double bond accounted for two of the six degrees of unsaturation, the remaining four degrees of unsaturation were ascribed to the presence of a tetracyclic compound.

By ^1^H–^1^H correlation spectroscopy (COSY) of **1**, proton signal correlations between δ_H_ 1.09/δ_H_ 1.84 and 1.49/δ_H_ 3.51/δ_H_ 2.31 and 2.22; δ_H_ 5.35/δ_H_ 1.99 and 1.49/δ_H_ 1.36/δ_H_ 1.06/δ_H_ 1.71 and 1.49/δ_H_ 3.46; δ_H_ 1.36/δ_H_ 0.92/δ_H_ 1.64 and 1.21/δ_H_ 1.76 and 1.52/δ_H_ 1.44/δ_H_ 1.77/δ_H_ 1.64 and 1.14/δ_H_ 2.12 and 1.92; δ_H_ 1.77/δ_H_ 1.04; δ_H_ 2.23/δ_H_ 1.02; and δ_H_ 2.23/δ_H_ 1.03 established the proton sequences H_2_-1/H_2_-2/ H-3/H_2_-4, H-6/H_2_-7/H-8/H-9/H_2_-11/H-12, H-8/H-14/H_2_-15/H_2_-16/H-17/H-20/H_2_-22/H_2_-23, H-20/H_3_-21, H-25/H_3_-26, and H-25/H_3_-27, respectively (Table 1). These data, together with the key heteronuclear multiple bond coherence (HMBC) correlations from H_2_-4 to C-5, C-6, and C-10; from H-6 to C-4 and C-10; from H_2_-7 to C-5; from H-8 to C-10; from H_2_-11 to C-13; from H-12 to C-18; from H-14 to C-12, C-13, and C-18; from H-17 to C-12, C-13, and C-18; from H_3_-18 to C-12, C-13, C-14, and C-17; from H_3_-19 to C-1, C-5, C-9, and C-10; from H_2_-23 to C-24 and C-28; from H-25 to C-23, C-24, and C-28; from H_3_-26 to C-24; from H_3_-27 to C-24; and from H_2_-28 to C-24 enabled elucidation of the carbon skeleton of **1** (Table 1). Moreover, the planar structure of **1** was determined by comparison of the ^13^C NMR data with those of a known principal sterol, 24-methylenecholesterol (**5**) (Figure 1) [5,6].

**Figure 2:**
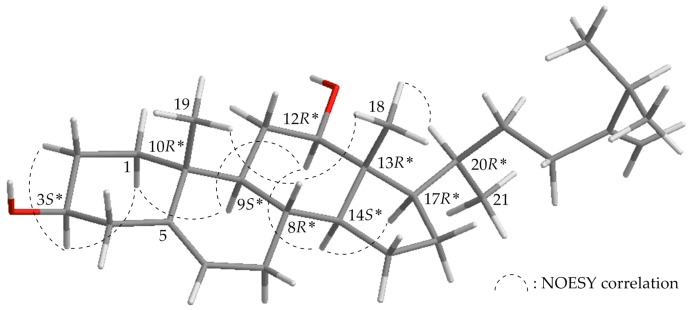
Computer-generated model of **1** using MM2 force field calculations and selected protons with key NOESY correlations. Red color: oxygen atom; gray color: hydrogen atom; black color: carbon atom; *: relative configuration.

The relative stereochemistry of **1** was established by comparison of NMR data with those of sterol **5** [5,6] and from the interactions observed in the nuclear Overhauser effect spectroscopy (NOESY) experiment, which were corroborated by MM2 force field calculations, suggesting the most stable conformation to be as shown in Figure 2 [7]. The configurations at C-3 (δ_C_ 71.7), C-8 (δ_C_ 30.6), C-9 (δ_C_ 49.5), C-10 (δ_C_ 36.5), C-13 (δ_C_ 47.6), C-14 (δ_C_ 54.7), C-17 (δ_C_ 57.3), and C-20 (δ_C_ 33.4) in **1** were found to be similar to those of **5** (C-3, δ_C_ 71.7; C-8, δ_C_ 31.9; C-9, δ_C_ 50.1; C-10, δ_C_ 36.5; C-13, δ_C_ 42.3; C-14, δ_C_ 56.7; C-17, δ_C_ 56.0; C-20, δ_C_ 35.7) [5], (C-3, δ_C_ 71.84; C-8, δ_C_ 31.94; C-9, δ_C_ 50.17; C-10, δ_C_ 36.52; C-13, δ_C_ 42.31; C-14, δ_C_ 56.80; C-17, δ_C_ 56.04; C-20, δ_C_ 35.79) [6]. A key correlation map obtained from the NOESY experiment for **1** showed interactions between H-1α (δ_H_ 1.84)/H-3 (δ_H_ 3.51), H-1α/H-9 (δ_H_ 1.06), H-2α (δ_H_ 1.84)/H-3, H-8 (δ_H_ 1.36)/H_3_-18 (δ_H_ 0.72), H-8/H_3_-19 (δ_H_ 1.02), H-9/H-12 (δ_H_ 3.46), H-12/H-14 (δ_H_ 0.92), H-14/H-17 (δ_H_ 1.44), and H_3_-18/H-20 (δ_H_ 1.77) (Figure 2). Thus, the hydroxyl groups at C-3 and C-12 should be positioned on the β-face according to modeling analysis [7]. The aforementioned findings clearly confirmed the structure of columnaristerol B (**1**).

**Figure 3:**
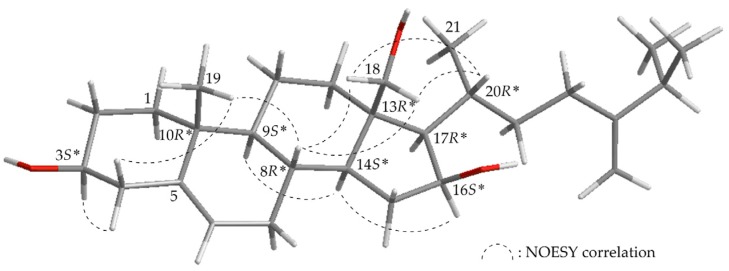
Computer-generated model of **2** using MM2 force field calculations and selected protons with key NOESY correlations. Red color: oxygen atom; gray color: hydrogen atom; black color: carbon atom; *: relative configuration.

HRESIMS of new metabolite columnaristerol C (**2**) suggested a molecular formula of C_28_H_46_O_3_, as analysis showed a signal at *m/z* 453.33366 (calcd. for C_28_H_46_O_3_ + Na, 453.33392); in addition, the presence of a hydroxyl group was determined, as the IR spectrum of **2** showed a band at ν_max_ 3331 cm^−1^. The existence of a tertiary methyl (δ_H_ 1.03), three secondary methyls (δ_H_ 1.04, 3H, d, *J* = 6.4 Hz; 1.02, 6H, d, *J* = 6.8 Hz), two oxymethines (δ_H_ 4.25, 1H, m; 3.53, 1H, m), and one oxymethylene (δ_H_ 4.00, 3.68, *J*_AB_ = 12.0 Hz) were identified from the ^1^H NMR data, and the presence of a trisubstituted double bond was revealed by NMR data at δ_H_ 5.38 (1H, m), and δ_C_ 140.9 (C) and 121.2 (CH) (Table 2). Combined analysis of the molecular formula and the resonances of the ^13^C NMR and DEPT spectra revealed that **2** contained 4 methyls, 10 sp^3^ methylenes (including 1 oxymethylene), 8 sp^3^ methines (including 2 oxymethines), 2 sp^3^ quaternary carbons, 1 sp^2^ methylene, 1 sp^2^ methine, and 2 sp^2^ quaternary carbons. Comparison of the NMR chemical shift values of **2** with those of 24-methylenecholesterol (**5**) [5,6], as well as its HMBC cross-peaks from H_2_-18 to C-12, C-13, C-14, and C-17, and ^1^H–^1^H COSY correlations between the oxymethine proton signal at δ_H_ 4.25 (H-16)/δ_H_ 2.42 and 1.52 (H_2_-15) and δ_H_ 4.25 (H-16)/δ_H_ 1.20 (H-17), suggested that **2** may be a 16,18-dihydroxyl analogue of **5**. Finally, the structure of **2** was confirmed based on the NOESY correlations (Figure 3) observed between H-3/H-4α, H-4β/H_3_-19, H-8/H_2_-18, H-8/H_3_-19, H-9/H-14, H-14/H-16, and H_2_-18/H-20.

Sterols **3** and **4** were identified as 5,6-epoxylitosterol (5β,6β-epoxyergost-24(28)-ene-3β,19-diol) and litosterol (ergosta-5,24(28)-diene-3β,19-diol), which had been previously isolated from the Okinawan soft coral *Litophyton viridis* [5]. To the best of our knowledge, this was the first time that these two marine-origin sterols had been obtained from *Nephthea columnaris*.

In vitro anti-inflammatory activity assays were performed using human neutrophils, and the results demonstrated that sterol **3** had inhibitory effects on the generation of superoxide anions and the release of elastase, with IC_50_ values of 4.60 and 3.90 μM, respectively, but sterol **4** displayed no anti-inflammatory effects (Table 3). Comparison with the structures and anti-inflammatory activities of sterols **3** and **4** implied that the presence of a 5β,6β-epoxy group enhanced the activity.

## 3. Experimental Section

### 3.1. General Experimental Procedures

Column chromatography was carried out using silica gel (230–400 mesh size; Merck, Darmstadt, Germany). TLC was performed on plates precoated with Kieselgel 60 (with fluorescent indicator 254, 0.25-mm-thick; Merck), and the spots on the plates were visualized by spraying with 10% H_2_SO_4_ solution followed by heating. Normal-phase HPLC (NP-HPLC) was performed using a HPLC system equipped with a Hitachi L-7110 pump (Hitachi, Tokyo, Japan) and an injection port (7725; Rheodyne, Rohnert Park, CA, USA). A semi-preparative normal-phase column (25 cm × 21.2 mm, 5 μM, Ascentis Si, Cat.: 581515-U, Sigma-Aldrich, St. Louis, MO, USA) was employed. Reverse-phase HPLC (RP-HPLC) was performed using a system equipped with a Hitachi L-7100 pump, a photodiode array detector (Hitachi L-2455), an injection port (Rheodyne 7725) and a 250 × 21.2 mm column (5 μM, Luna RP-18e; Phenomenex Inc., Torrance, CA, USA) or a 250 × 10.0 mm column (5 μM; Ascentis C18 Cat.: 581343-U, Sigma-Aldrich). IR spectra were obtained using a spectrophotometer (Nicolet iS5 FT-IR; Thermo Scientific, Waltham, MA, USA). NMR spectra were recorded on a NMR spectrometer (Varian Mercury 400 MHz Plus system; Varian, Palo Alto, CA, USA) using the residual CHCl_3_ signal (δ_H_ 7.26 ppm) and CDCl_3_ signal (δ_C_ 77.1 ppm) as the internal standard for ^1^H NMR and ^13^C NMR, respectively. Coupling constants (*J*) are presented in Hz. ESIMS and HRESIMS were recorded using a mass spectrometer (7 Tesla SolariX FTMS system; Bruker, Bremen, Germany). Melting points of the natural products were determined using a Fargo apparatus (Panchum Scientific, Kaohsiung, Taiwan), and the values were uncorrected. Optical rotation values were measured using a digital polarimeter (Jasco P-1010; Japan Spectroscopic, Tokyo, Japan).

### 3.2. Animal Material

Specimens of the octocoral *N. columnaris* were collected by hand using scuba equipment off the coast of Southern Taiwan in February, 2012. The samples were stored in a −20 °C freezer immediately until extraction. A voucher specimen (NMMBA-TW-SC-12057) was deposited in the National Museum of Marine Biology and Aquarium, Taiwan [8].

### 3.3. Extraction and Isolation

Sliced bodies of *N. columnaris* (wet weight 800 g, dry weight 76.7 g) were extracted with a mixture of methanol (MeOH) and dichloromethane (DCM) (*v:v* = 1:1). The extract (25.1 g) was partitioned between ethyl acetate (EtOAc) and H_2_O. The EtOAc layer (7.35 g) was separated on silica gel and eluted with *n*-hexane/EtOAc (stepwise, *v:v* = 100:1 to 100% EtOAc) to yield 12 subfractions A–L. Fraction G was separated by silica gel column chromatography and then eluted with *n*-hexane/acetone (stepwise, *v:v* = 20:1 to 100% acetone) to afford 10 subfractions G1–G10. Fraction G5 was purified by NP-HPLC using a mixture of *n*-hexane/acetone (*v:v* = 3:1) to afford ten subfractions G5A–G5J. Fraction G5E was repurified by RP-HPLC using a mixture of acetonitrile/H_2_O (*v:v* = 80:20) to yield four subfractions G5E1–G5E4. Fraction G5E3 was repurified by RP-HPLC using a mixture of MeOH/H_2_O (*v:v* = 95:5 at a flow rate of 4.0 mL/min) to yield **1** (0.8 mg). Fraction I was separated by silica gel column chromatography and then eluted with *n*-hexane/EtOAc (stepwise, *v:v* = 100:1 to 100% EtOAc) to afford 15 subfractions I1–I15. Fraction I13 was purified by NP-HPLC using a mixture of *n*-hexane/acetone/DCM (*v:v:v* = 3:1:1) to afford 11 subfractions I13A–I13K. Fraction I13G was repurified by RP-HPLC using a mixture of MeOH/H_2_O/tetrahydrofuran (*v:v:v* = 90:9.5:0.5 at a flow rate of 2.0 mL/min) to yield **2** (0.7 mg). Fraction I13H was repurified by RP-HPLC using a mixture of MeOH/H_2_O (*v:v* = 80:20) to afford seven subfractions I13H1–I13H7. Fraction I13H7 was further separated by NP-HPLC using a mixture of *n*-hexane/EtOAC (*v:v* = 2:1) to afford three subfractions I13H7A–I13H7C. Fraction I13H7C was repurified by RP-HPLC using a mixture of MeOH/H_2_O (*v:v* = 95/5 at a flow rate of 4.0 mL/min) to yield **3** (1.3 mg) and **4** (5.1 mg), respectively.

Columnaristerol B (**1**): white powder; mp 143–144 °C; $\alpha_{D}^{25}$ −200 (*c* 0.53, CHCl_3_); IR (neat) ν_max_ 3386 cm^−1^, 1643 cm^−1^; ^1^H (400 MHz, CDCl_3_) and ^13^C (100 MHz, CDCl_3_) NMR data (see Table 1); ESIMS: *m*/*z* 437 [M + Na]^+^; HRESIMS: *m*/*z* 437.33918 (calcd. for C_28_H_46_O_2_ + Na, 437.33900).

Columnaristerol C (**2**): white powder; mp 140 °C; $\alpha_{D}^{25}$ +31 (*c* 0.22, CHCl_3_); IR (neat) ν_max_ 3331 cm^−1^, 1640 cm^−1^; ^1^H (400 MHz, CDCl_3_) and ^13^C (100 MHz, CDCl_3_) NMR data (see Table 2); ESIMS: *m*/*z* 453 [M + Na]^+^; HRESIMS: *m*/*z* 453.33366 (calcd. for C_28_H_46_O_3_ + Na, 453.33392).

5,6-Epoxylitosterol (**3**): white powder; mp 160–161 °C (ref. [5], mp 179–183 °C); $\alpha_{D}^{25}$ +17 (*c* 0.07, CHCl_3_) (ref. [5], [α] D +3.8 (*c* 0.26, CHCl3)); IR (neat) ν_max_ 3386 cm^−1^, 1641 cm^−1^; ^1^H (CDCl_3_, 400 MHz) and ^13^C (CDCl_3_, 100 MHz) NMR data were found to be in complete agreement with previous reports [5]; ESIMS *m/z* 453 [M + Na]^+^.

Litosterol (**4**): white powder; mp 147–149 °C (ref. [5], mp 147.5–150 °C); $\alpha_{D}^{25}$ −31 (*c* 0.26, CHCl_3_) (ref. [5], [α] D −25.8 (*c* 0.24, CHCl_3_)); IR (neat) ν_max_ 3419 cm^−1^, 1635 cm^−1^; ^1^H (CDCl_3_, 400 MHz) and ^13^C (CDCl_3_, 100 MHz) NMR data were found to be in complete agreement with previous reports [5]; ESIMS *m/z* 437 [M + Na]^+^

### 3.4. Molecular Mechanics Calculations

Implementation of the MM2 force field [7] in ChemBio 3D Ultra software from Cambridge Soft Corporation (ver. 12.0, Cambridge, MA, USA) was used to create molecular models.

### 3.5. Generation of Superoxide Anions and Release of Elastase by Human Neutrophils

Human neutrophils were obtained from whole blood using the method of dextran sedimentation and Ficoll centrifugation. Measurements of superoxide anion generation and the release of elastase by neutrophils were carried out according to previously described procedures [9,10]. Briefly, superoxide anion production was assayed by monitoring the superoxide dismutase-inhibitable reduction of ferricytochrome *c*. Elastase release experiments were performed using MeO–Suc–Ala–Ala–Pro–Valp–nitroanilide as the elastase substrate.

## 4. Conclusions

In the present study, our further investigation of natural substances obtained by extraction of *N. columnaris* led to the isolation of two new sterols, columnaristerols B (**1**) and C (**2**), as well as two known sterols, 5,6-epoxylitosterol (**3**) and litosterol (**4**) [5]. Our results demonstrated that **3** possessed potential anti-inflammatory activity in a human neutrophil model. The findings suggested that continued investigation of interesting secondary metabolites together with potentially useful bioactive substances from *N. columnaris* is worthwhile to inform potential new drug development.

## Figures and Tables

**Table 1:** ^1^H (400 MHz, CDCl_3_) and ^13^C (100 MHz, CDCl_3_) NMR data and ^1^H–^1^H COSY and HMBC correlations for sterol **1**.

Position	δ_H_ (*J* in Hz)	δ_C_, Multiple	^1^H–^1^H COSY	HMBC
1α/β	1.84 m; 1.09 m	37.2, CH_2_	H_2_-2	C-2, C-3, C-5, C-10, C-19
2α/β	1.84 m; 1.49 m	31.6, CH_2_	H_2_-1, H-3	C-3
3	3.51 m	71.7, CH	H_2_-2, H_2_-4	n. o. ^a^
4a/b	2.31 ddd (12.8, 4.8, 2.0); 2.22 m	42.1, CH_2_	H-3	C-2, C-3, C-5, C-6, C-10
5	-	140.6, C	-	-
6	5.35 br d (5.2)	121.6, CH	H_2_-7	C-4, C-7, C-8, C-10
7a/b	1.99 m; 1.49 m	31.5, CH_2_	H-6, H-8	C-5, C-6, C-8, C-9
8	1.36 m	30.6, CH	H_2_-7, H-9, H-14	C-7, C-9, C-10, C-13, C-14
9	1.06 m	49.5, CH	H-8, H_2_-11	C-7, C-11
10	-	36.5, C	-	-
11a/b	1.71 m; 1.49 m	31.3, CH_2_	H-9, H-12	C-8, C-9, C-12, C-13
12	3.46 ddd (10.8, 5.2, 4.4)	79.5, CH	H_2_-11	C-18
13	-	47.6, C	-	-
14	0.92 m	54.7, CH	H-8, H_2_-15	C-8, C-12, C-13, C-15, C-18
15a/b	1.64 m; 1.21 m	23.8, CH_2_	H-14, H_2_-16	C-8, C-14, C-16, C-17
16a/b	1.76 m; 1.52 m	24.4, CH_2_	H_2_-15, H-17	n. o.
17	1.44 m	57.3, CH	H_2_-16, H-20	C-12, C-13, C-16, C-18, C-20, C-21, C-22
18	0.72 s	7.8, CH_3_	-	C-12, C-13, C-14, C-17
19	1.02 s	19.3, CH_3_	-	C-1, C-5, C-9, C-10
20	1.77 m	33.4, CH	H-17, H_3_-21, H_2_-22	C-16
21	1.04 d (6.4)	21.5, CH_3_	H-20	C-17
22a/b	1.64 m; 1.14 m	33.4, CH_2_	H-20, H_2_-23	C-21
23a/b	2.12 m; 1.92 m	32.5, CH_2_	H_2_-22	C-20, C-22, C-24, C-28
24	-	156.8, C	-	-
25	2.23 m	33.8, CH	H_3_-26, H_3_-27	C-23, C-24, C-26, C-27, C-28
26	1.02 d (6.8)	21.8, CH_3_	H-25	C-24, C-25, C-27
27	1.03 d (6.8)	22.0, CH_3_	H-25	C-24, C-25, C-26
28a/b	4.72 br s; 4.67 br s	106.0, CH_2_	-	C-23, C-24, C-25

^a^ n. o. = not observed.

**Table 2:** ^1^H (400 MHz, CDCl_3_) and ^13^C (100 MHz, CDCl_3_) NMR data and ^1^H–^1^H COSY and HMBC correlations for sterol **2**.

Position	δ_H_ (*J* in Hz)	δ_C_, Multiple	^1^H–^1^H COSY	HMBC
1a/b	1.81 m, 1.01 m	37.2, CH_2_	H_2_-2	C-5
2a/b	1.83 m, 1.48 m	31.6, CH_2_	H_2_-1, H-3	n. o. ^a^
3	3.53 m	71.7, CH	H_2_-2, H_2_-4	n. o.
4α/β	2.32 m, 2.21 m	42.2, CH_2_	H-3	C-3, C-5, C-6
5	-	140.9, C	-	-
6	5.38 m	121.2, CH	H_2_-7	C-4, C-7, C-8, C-10
7a/b	2.30 m, 1.69 m	31.0, CH_2_	H-6, H-8	C-5, C-6
8	1.94 m	27.6, CH	H_2_-7, H-9, H-14	C-14
9	1.08 m	50.5, CH	H-8, H_2_-11	C-19
10	-	36.6, C	-	-
11	1.50 m	21.4, CH_2_	H-9, H_2_-12	n. o.
12a/b	2.12 m, 1.17 m	38.9, CH_2_	H_2_-11	C-18
13	-	46.4, C	-	-
14	1.14 m	60.4, CH	H-8, H_2_-15	C-8, C-9, C-12, C-13, C-16, C-17, C-18
15a/b	2.42 m, 1.50 m	41.8, CH_2_	H-14, H-16	C-13, C-16, C-17
16	4.25 m	70.1, CH	H_2_-15, H-17	C-13, C-17
17	1.20 m	55.8, CH	H-16, H-20	C-13, C-15, C-18, C-20
18a/b	4.00 d (12.0), 3.68 d (12.0)	62.8, CH_2_	-	C-12, C-13, C-14, C-17
19	1.03 s	19.4, CH_3_	-	C-1, C-5, C-9, C-10
20	1.95 m	34.9, CH	H-17, H_3_-21, H_2_-22	C-22
21	1.04 d (6.4)	19.2, CH_3_	H-20	C-17, C-20, C-22
22a/b	1.55 m, 1.17 m	34.9, CH_2_	H-20, H_2_-23	n. o.
23a/b	2.10 m, 1.91 m	30.7, CH_2_	H_2_-22	C-20, C-22, C-24, C-28
24	-	156.6, C	-	-
25	2.21 m	33.8, CH	H_3_-26, H_3_-27	C-23, C-24, C-26, C-27, C-28
26	1.02 d (6.8)	21.9, CH_3_	H-25	C-24, C-27
27	1.02 d (6.8)	22.0, CH_3_	H-25	C-24, C-26
28a/b	4.72 br s, 4.66 br s	106.0, CH_2_	-	C-23, C-25

^a^ n. o. = not observed.

**Table 3:** Inhibitory effects of sterols **1**–**4** on superoxide anion generation and elastase release by human neutrophils in response to fMet-Leu-Phe/Cytochalastin B.

Compound	Superoxide Anions	Elastase Release
	IC_50_ (μM) ^a^	IC_50_ (μM)
**1**	>10	>10
**2**	>10	>10
**3**	4.60 ± 0.85	3.90 ± 0.88
**4**	>10	>10
**LY294002 ^b^**	1.94 ± 0.81	4.44 ± 0.72

^a^ Concentration necessary for 50% inhibition (IC_50_); results are presented as mean ± SEM (*n* = 3). ^b^ LY294002 (2-morpholin-4-yl-8-phenylchromen-4-one) was used as the reference compound.

## Supplementary Materials

HRESIMS, and 1D (^1^H NMR, ^13^C NMR, and DEPT spectra) and 2D (HSQC, ^1^H−^1^H COSY, HMBC, and NOESY spectra) NMR spectra of new compounds **1** and **2** are available online at www.mdpi.com/1660-3397/15/7/212/s1.
