# Supplementary material for: Sterols from the Octocoral Nephthea columnaris
Source: Mar Drugs. 2017 Jul 4;15(7):212. doi: 10.3390/md15070212 (PMC5532654; doi:10.3390/md15070212)
Supplement: Supplementary file 1 [file marinedrugs-15-00212-s001.pdf]

Supporting Information

No	Content	page
S01.	HRESIMS spectrum of compound **1**.	2
S02.	^{1}H NMR spectrum (400 MHz) of compound **1** in $CDCl_3$.	2
S03.	^{13}C NMR spectrum (100 MHz) of compound **1** in $CDCl_3$.	3
S04.	^{13}C NMR (0–40 ppm) spectrum (100 MHz) of compound **1** in $CDCl_3$.	3
S05.	^{13}C NMR (40−80 ppm)spectrum (100 MHz) of compound **1** in $CDCl_3$.	4
S06.	^{13}C NMR (100–160 ppm) spectrum (100 MHz) of compound **1** in $CDCl_3$.	4
S07.	DEPT spectrum of compound **1** in $CDCl_3$.	5
S08.	DEPT (0–40 ppm) spectrum of compound **1** in $CDCl_3$.	5
S09.	DEPT (40−80 ppm) spectrum of compound **1** in $CDCl_3$.	6
S10.	DEPT (100–160 ppm) spectrum of compound **1** in $CDCl_3$.	6
S11.	gHSQC spectrum of compound **1** in $CDCl_3$.	7
S12.	^{1}H–^{1}H COSY spectrum of compound **1** in $CDCl_3$.	7
S13.	gHMBC spectrum of compound **1** in $CDCl_3$.	8
S14.	NOESY spectrum of compound **1** in $CDCl_3$.	8
S15.	HRESIMS spectrum of compound **2**.	9
S16.	^{1}H NMR spectrum (400 MHz) of compound **2** in $CDCl_3$.	9
S17.	^{13}C NMR spectrum (100 MHz) of compound **2** in $CDCl_3$.	10
S18.	^{13}C NMR (15–40 ppm) spectrum (100 MHz) of compound **2** in $CDCl_3$.	10
S19.	^{13}C NMR (40–80 ppm) spectrum (100 MHz) of compound **2** in $CDCl_3$.	11
S20.	^{13}C NMR (100–160 ppm) spectrum (100 MHz) of compound **2** in $CDCl_3$.	11
S21.	DEPT spectrum of compound **2** in $CDCl_3$.	12
S22.	DEPT (15–40 ppm) spectrum of compound **2** in $CDCl_3$.	12
S23.	DEPT (40–80 ppm) spectrum of compound **2** in $CDCl_3$.	13
S24.	DEPT (100–160 ppm) spectrum of compound **2** in $CDCl_3$.	13
S25.	gHSQC spectrum of compound **2** in $CDCl_3$.	14
S26.	^{1}H–^{1}H COSY spectrum of compound **2** in $CDCl_3$.	14
S27.	gHMBC spectrum of compound **2** in $CDCl_3$.	15
S28.	NOESY spectrum of compound **2** in $CDCl_3$.	15

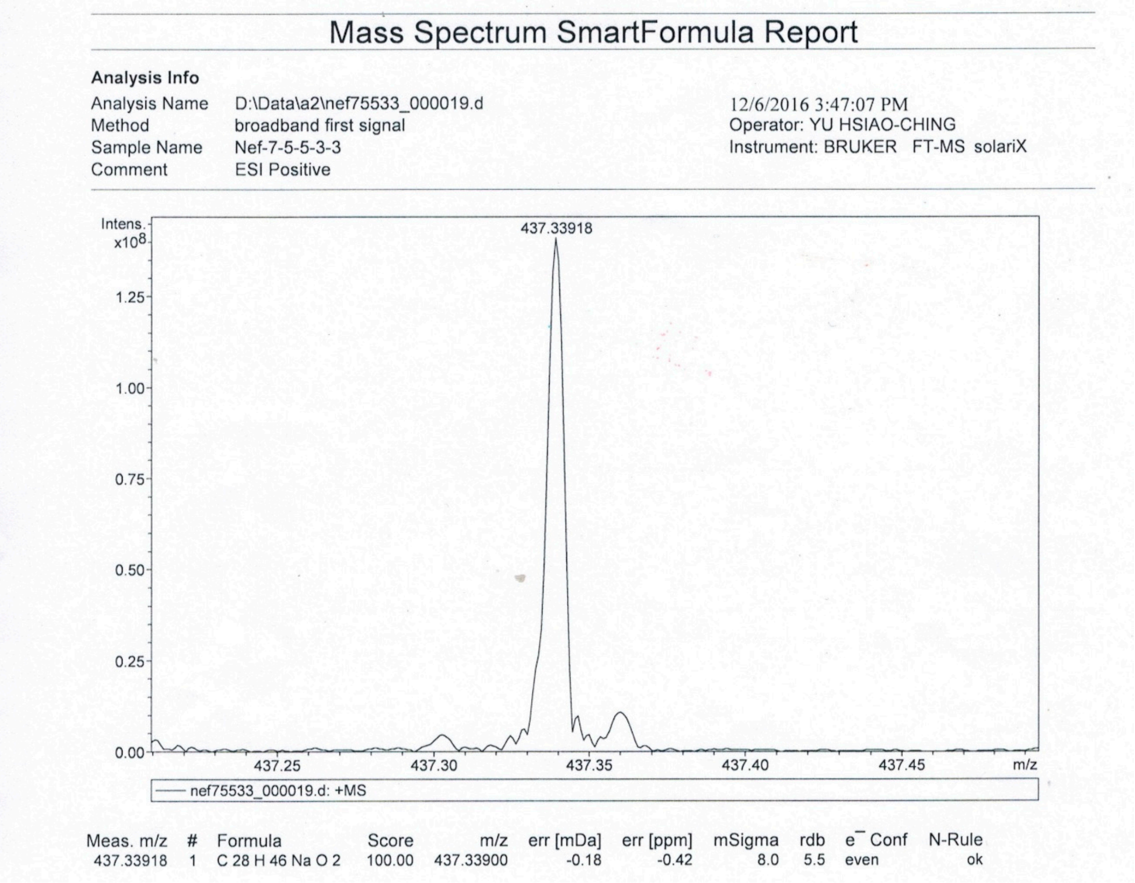

S01. HRESIMS spectrum of compound **1**.

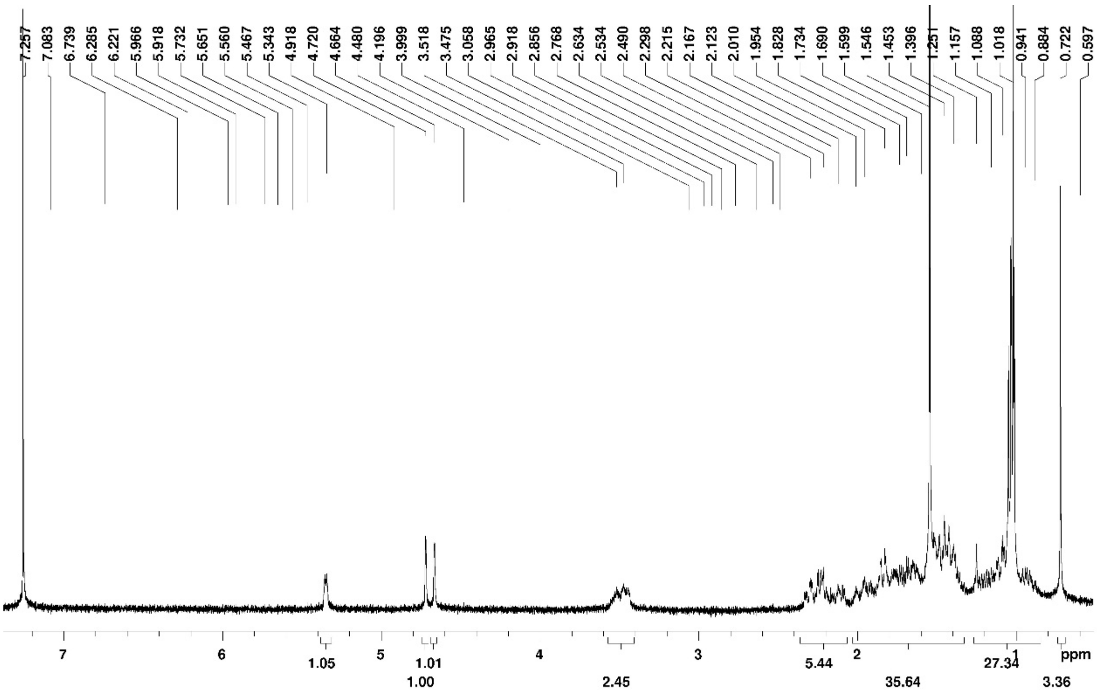

S02. ^{1}H NMR spectrum (400 MHz) of compound **1** in $CDCl_3$.

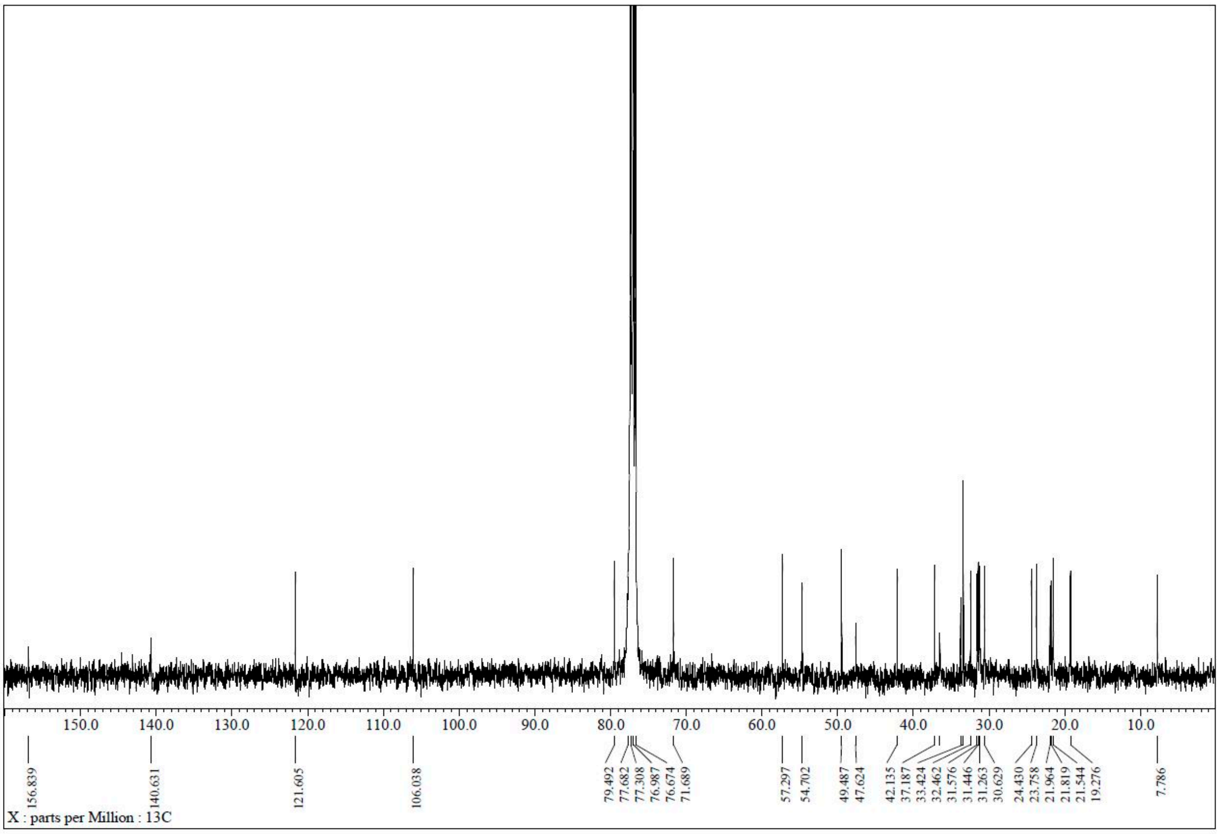

S03. ^{13}C NMR spectrum (100 MHz) of compound **1** in $CDCl_3$.

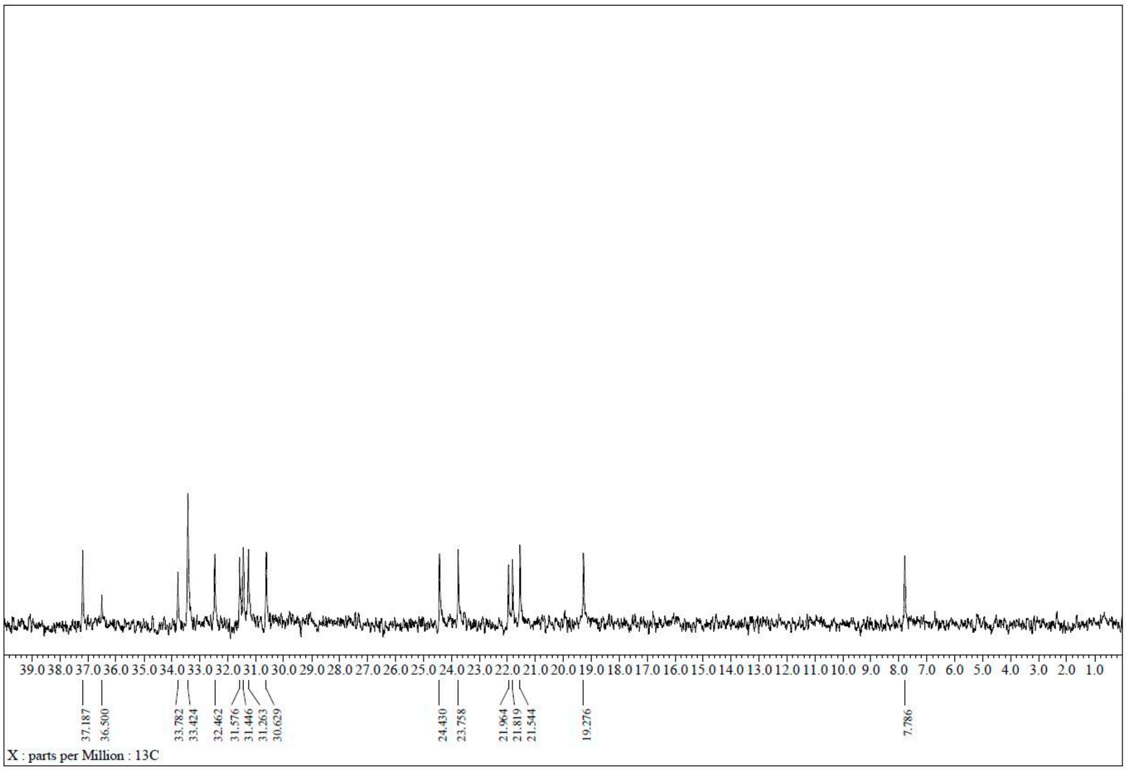

S04. ^{13}C NMR (0–40 ppm) spectrum (100 MHz) of compound **1** in $CDCl_3$.

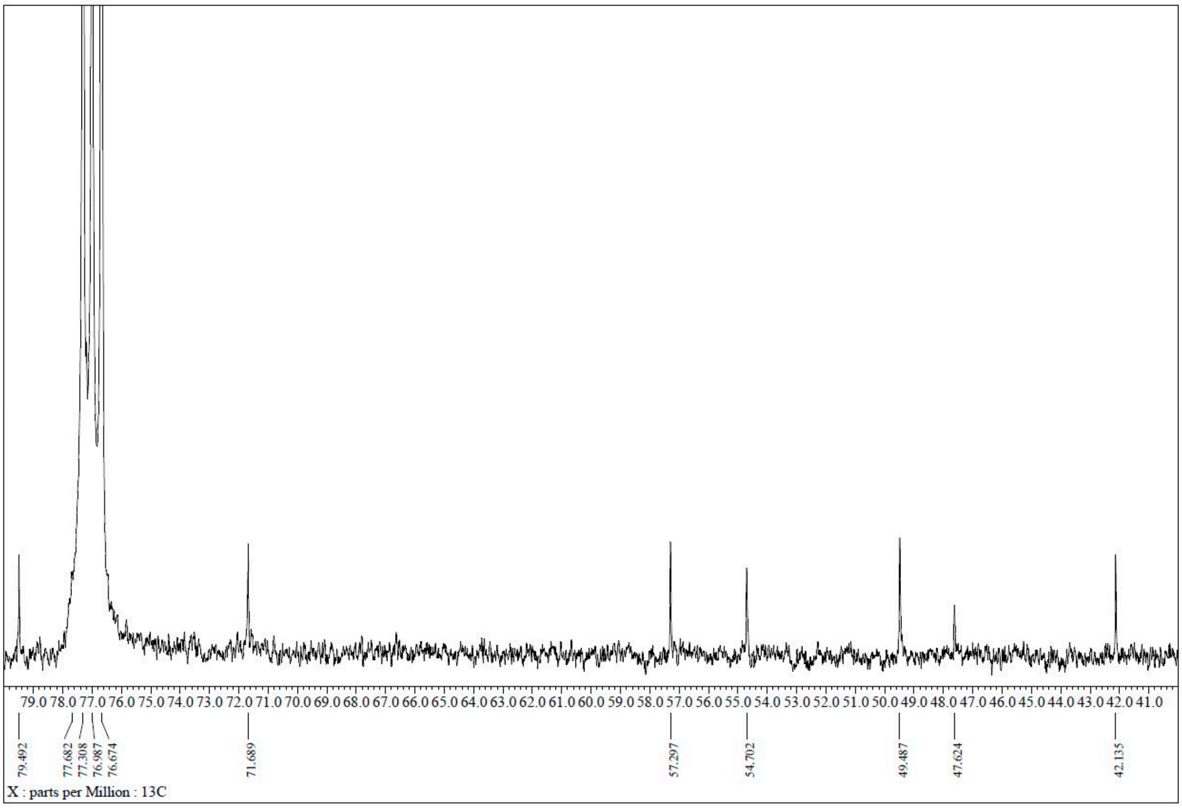

S05. ^{13}C NMR (40−80 ppm) spectrum (100 MHz) of compound **1** in $CDCl_3$.

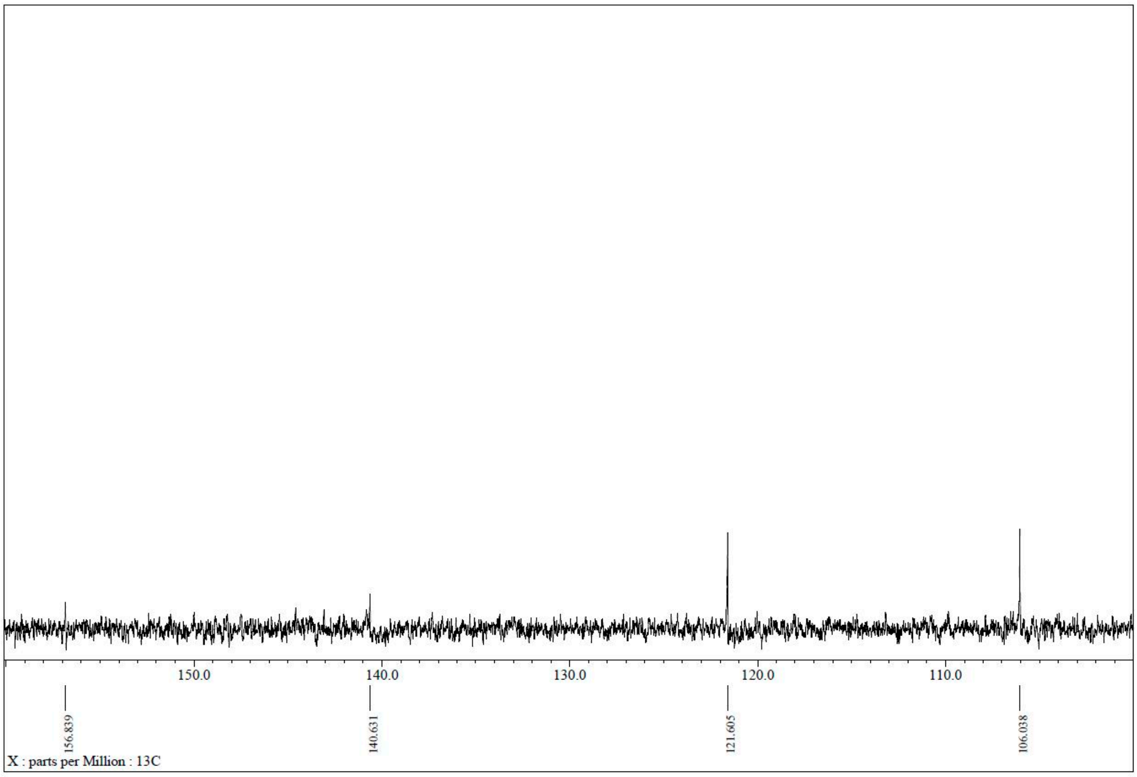

S06. ^{13}C NMR (100−160 ppm) spectrum (100 MHz) of compound **1** in $CDCl_3$.

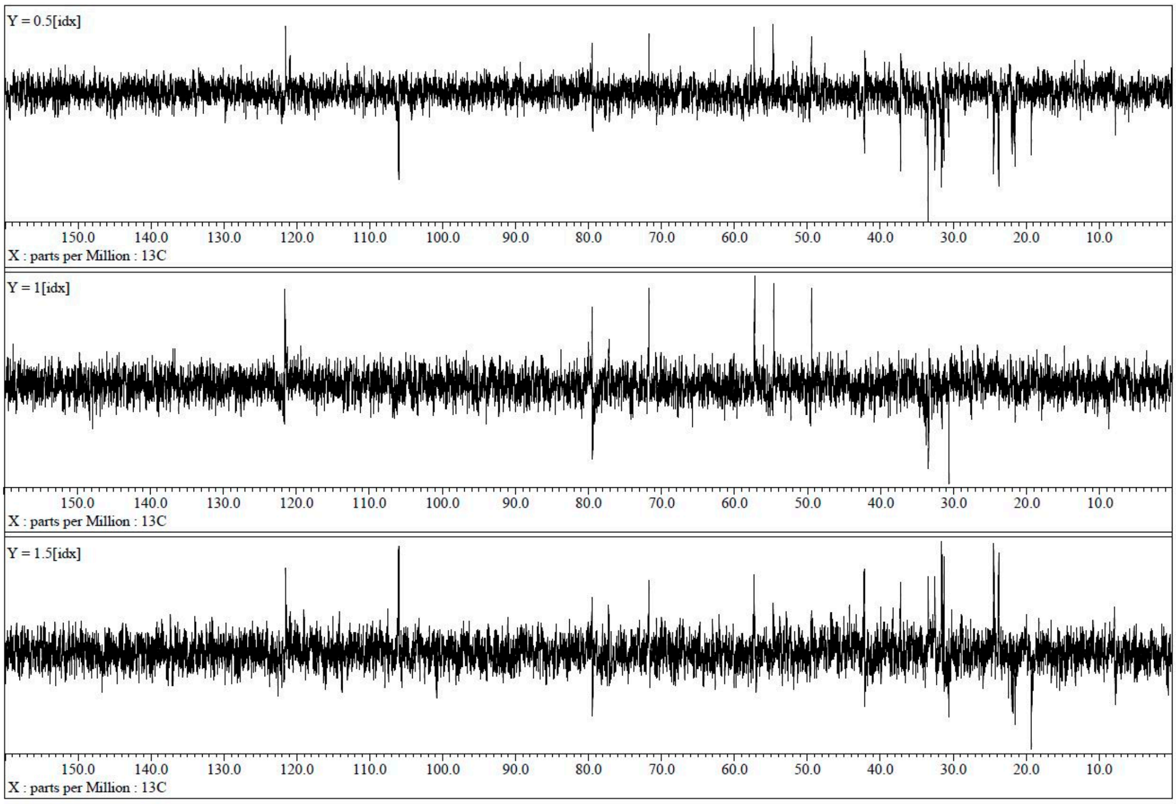

S07. DEPT spectrum of compound **1** in $CDCl_3$.

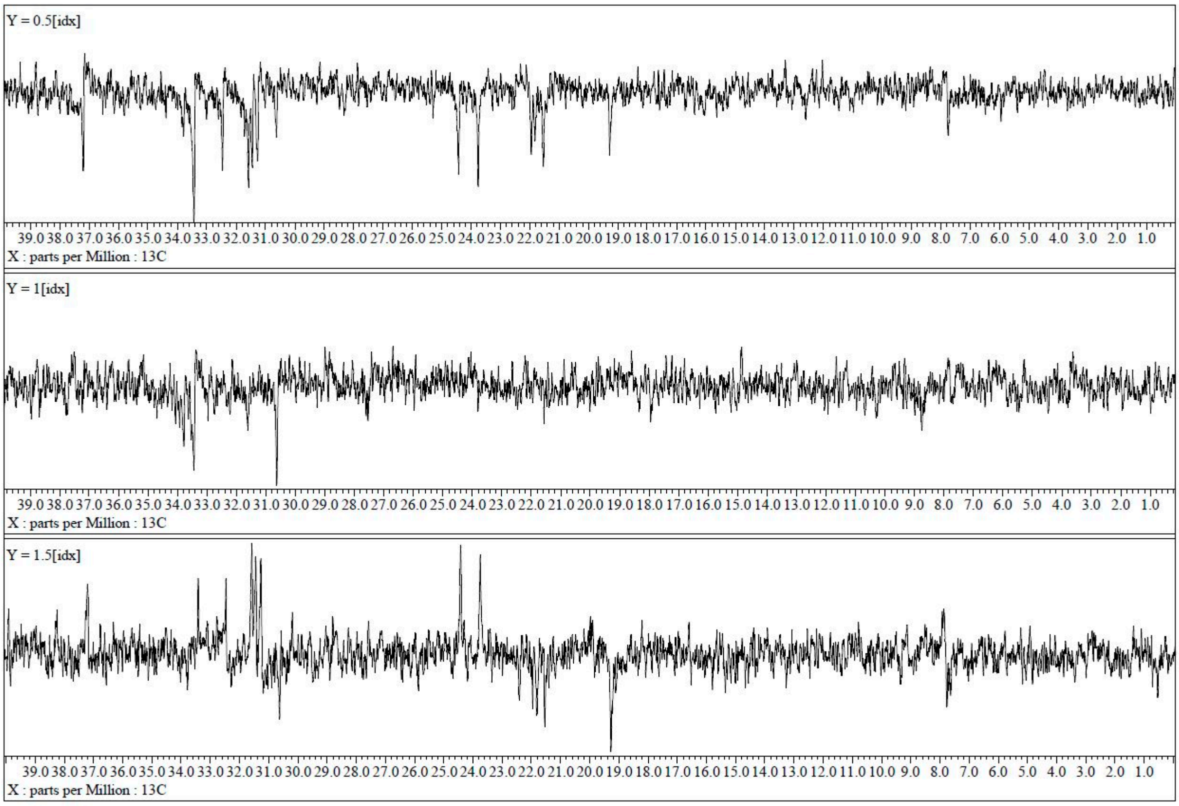

S08. DEPT (0–40 ppm) spectrum of compound **1** in $CDCl_3$.

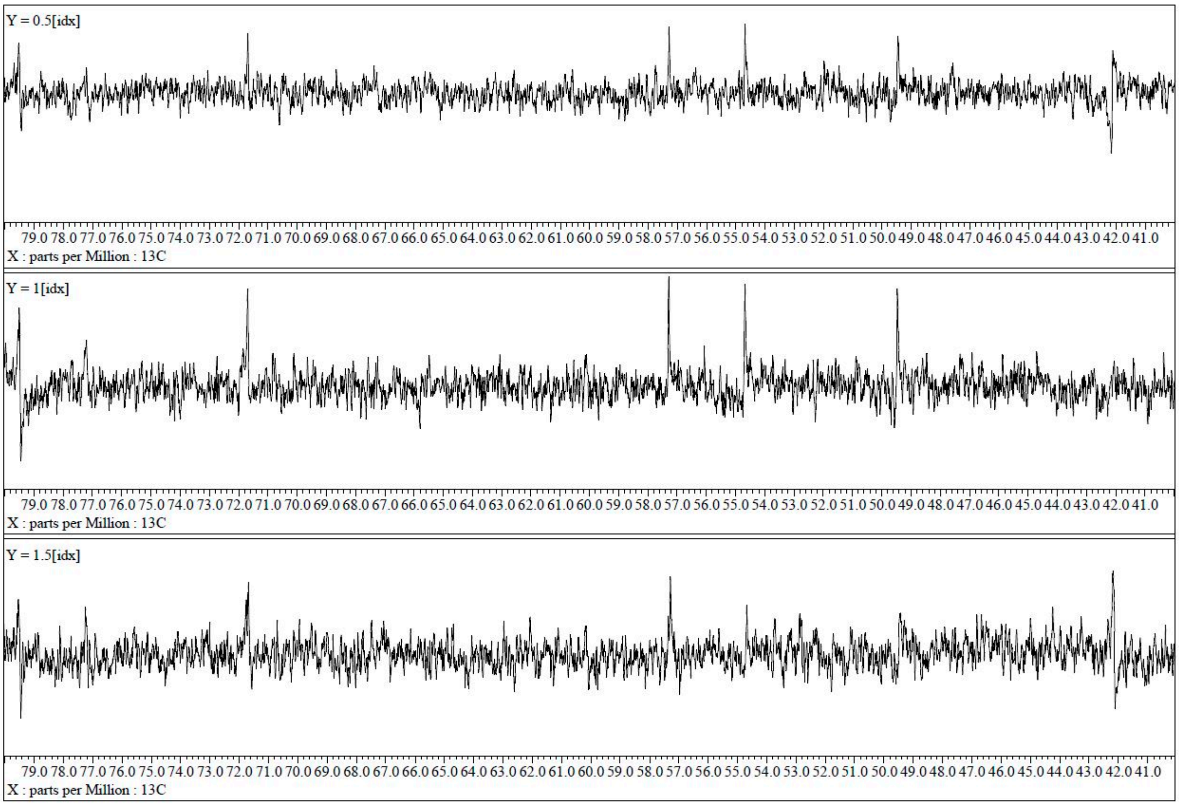

S09. DEPT (40−80 ppm) spectrum of compound **1** in $CDCl_3$.

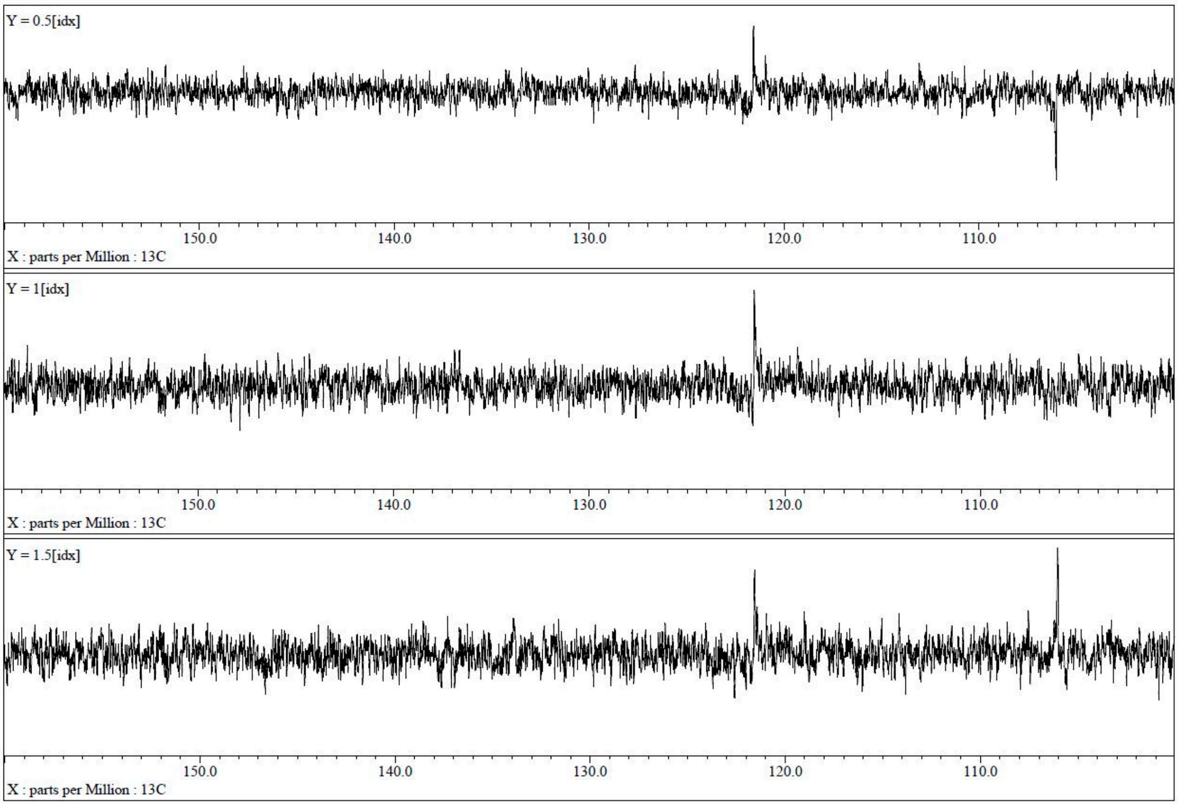

S10. DEPT (100−160 ppm) spectrum of compound **1** in $CDCl_3$.

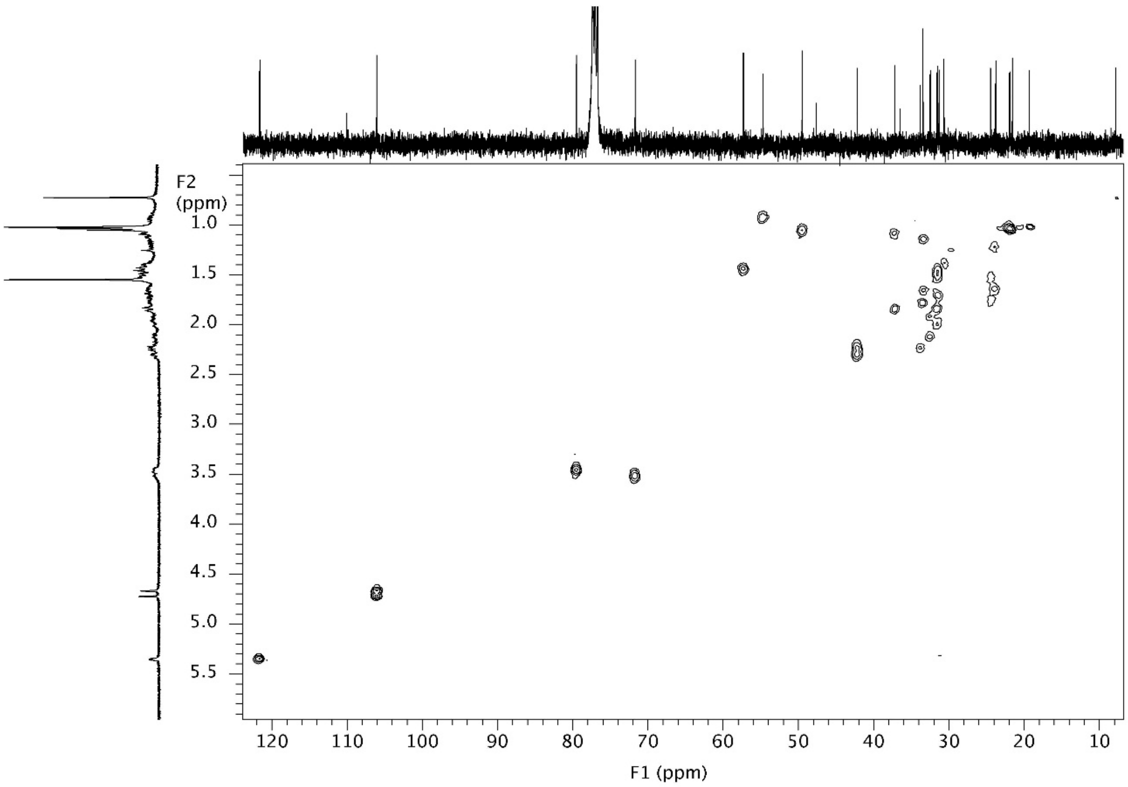

S11. gHSQC spectrum of compound **1** in $CDCl_3$.

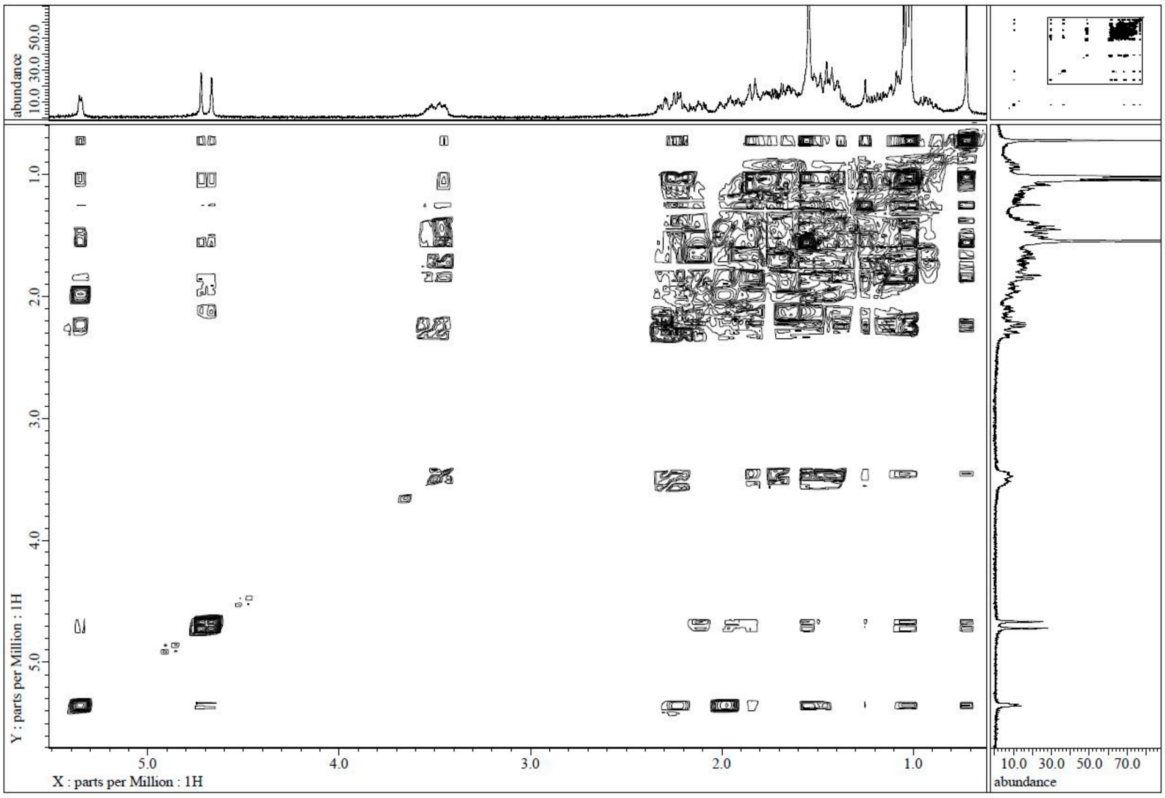

S12. ^1H–^1H COSY spectrum of compound **1** in $CDCl_3$.

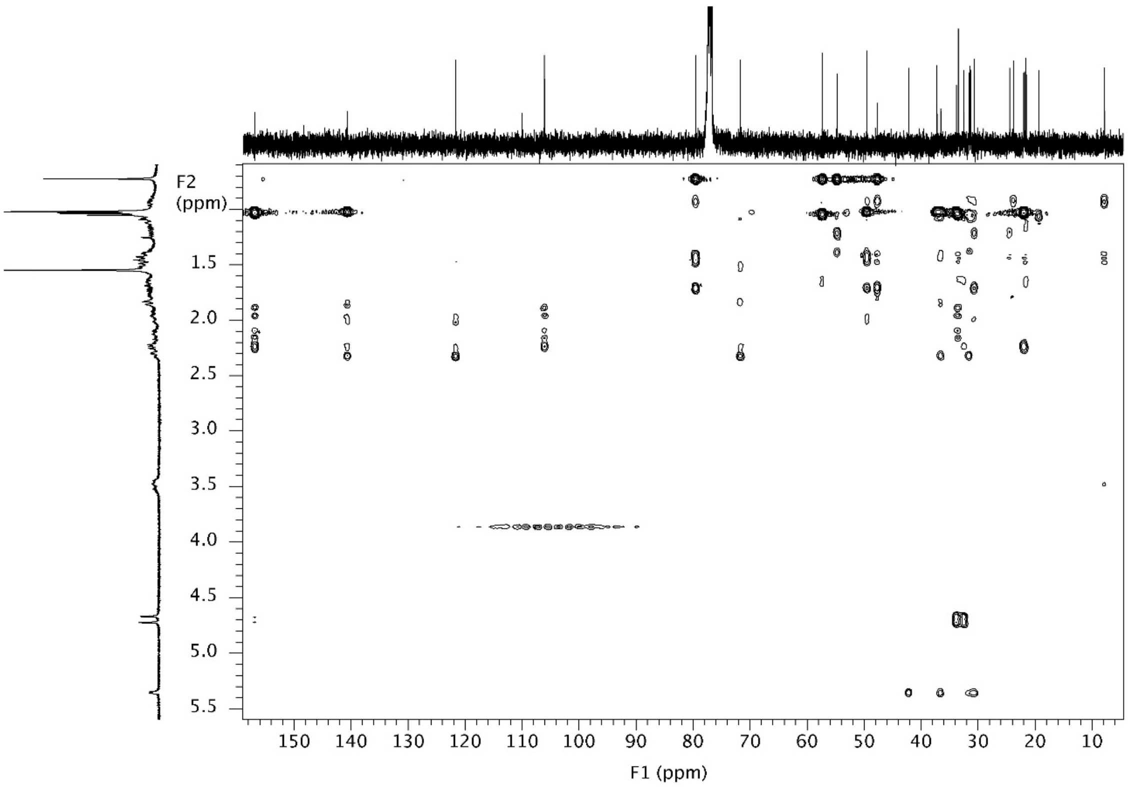

S13. gHMBC spectrum of compound **1** in $CDCl_3$.

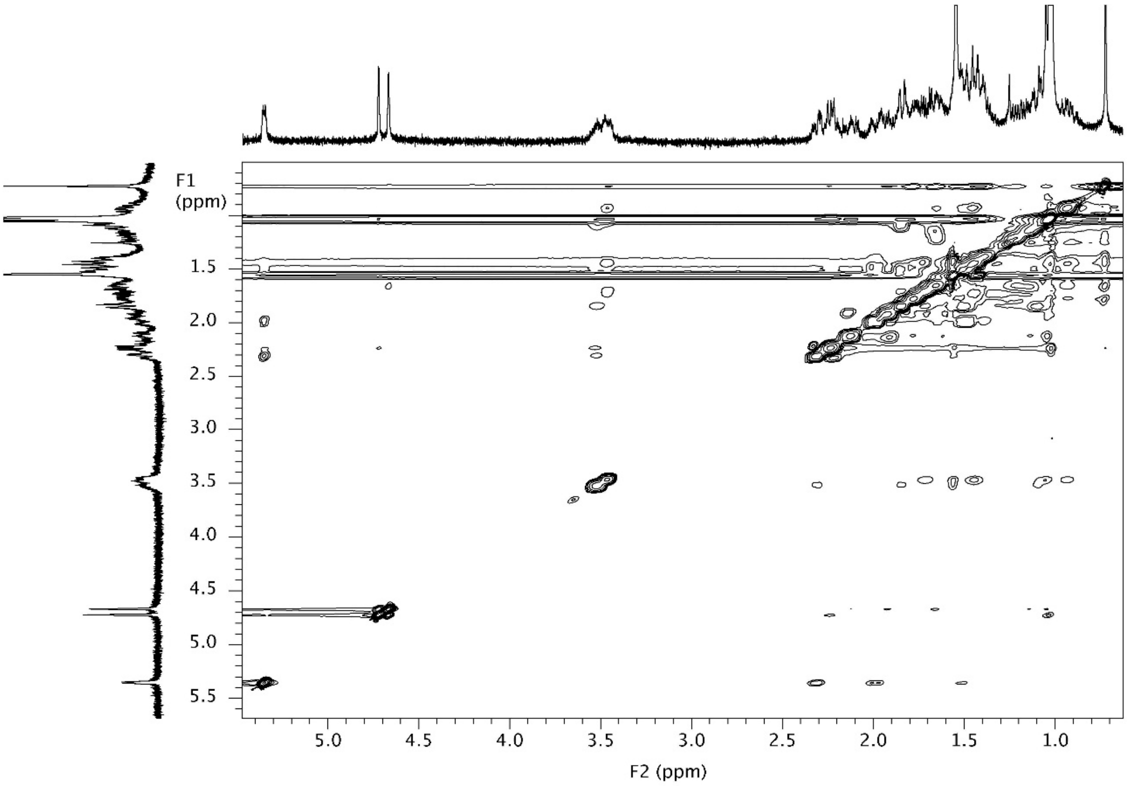

S14. NOESY spectrum of compound **1** in $CDCl_3$.

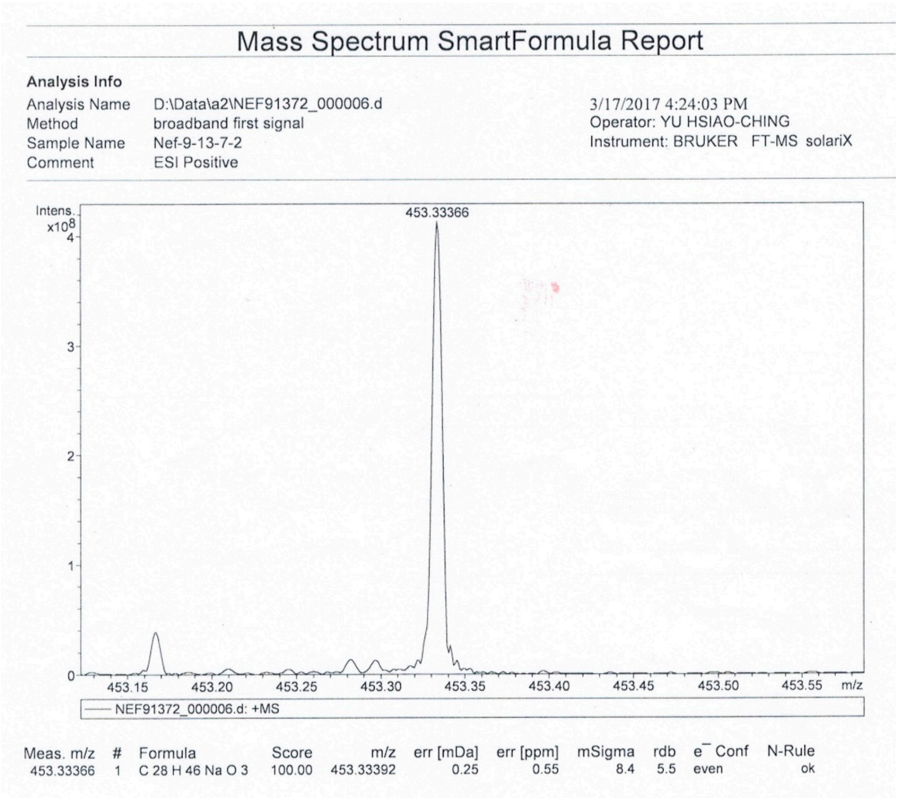

S15. HRESIMS spectrum of compound **2**.

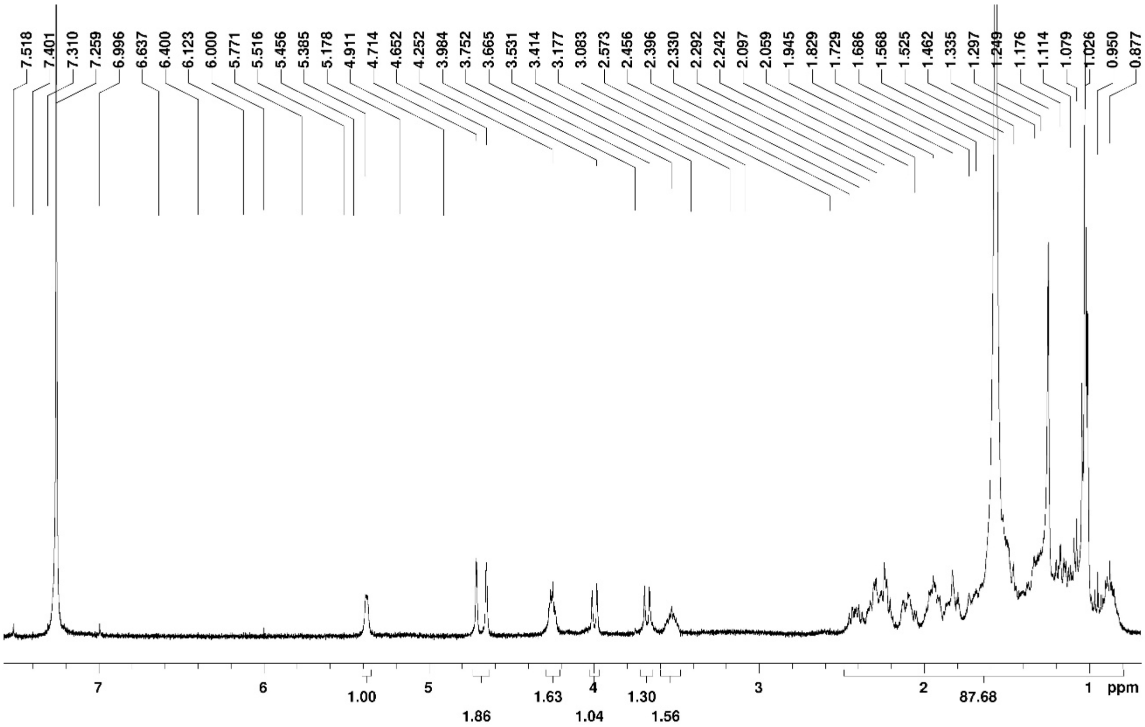

S16. ^{1}H NMR spectrum (400 MHz) of compound **2** in $CDCl_3$.

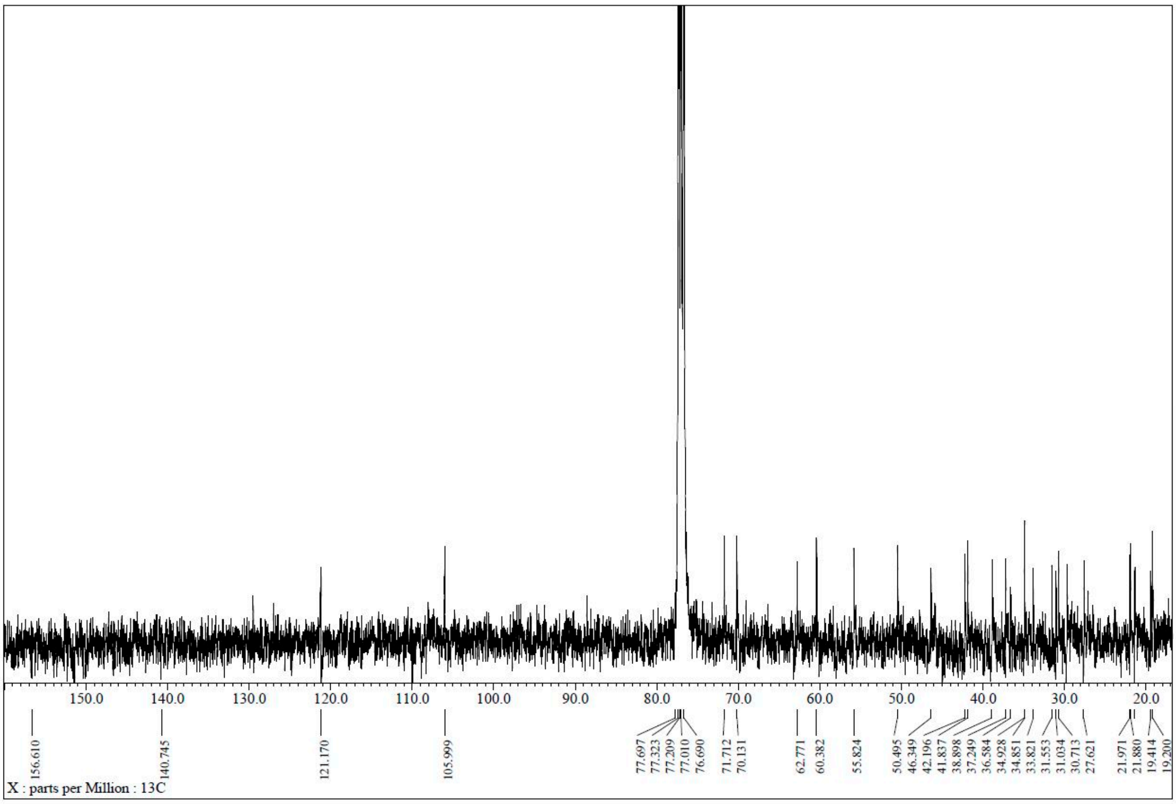

S17. ^{13}C NMR spectrum (100 MHz) of compound **2** in $CDCl_3$.

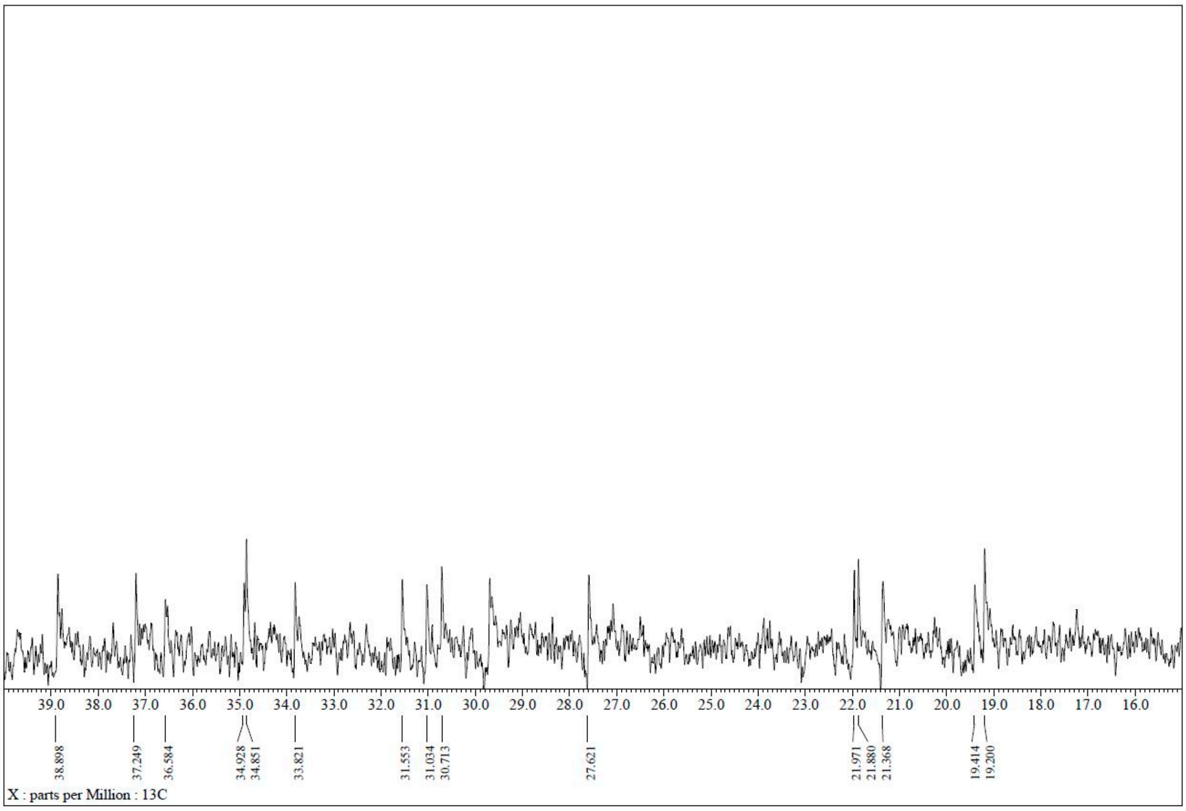

S18. ^{13}C NMR (15–40 ppm) spectrum (100 MHz) of compound **2** in $CDCl_3$.

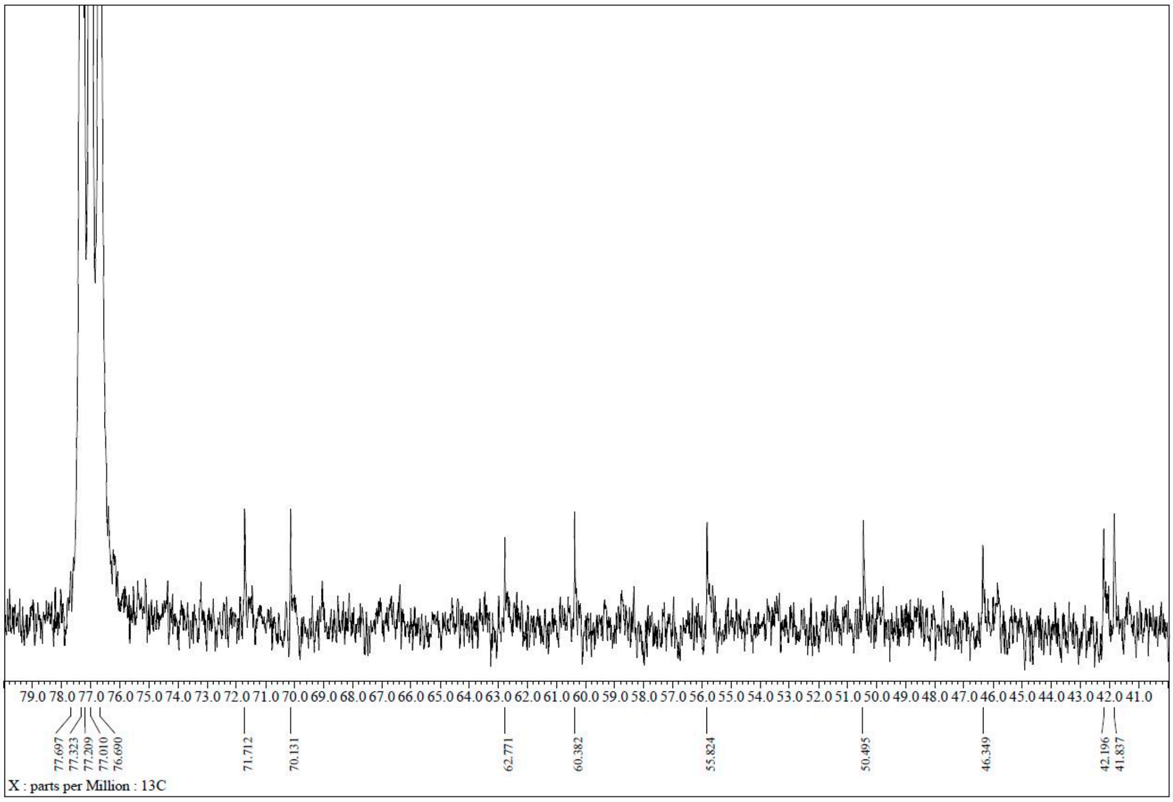

S19. ^{13}C NMR (40−80 ppm) spectrum (100 MHz) of compound **2** in $CDCl_3$.

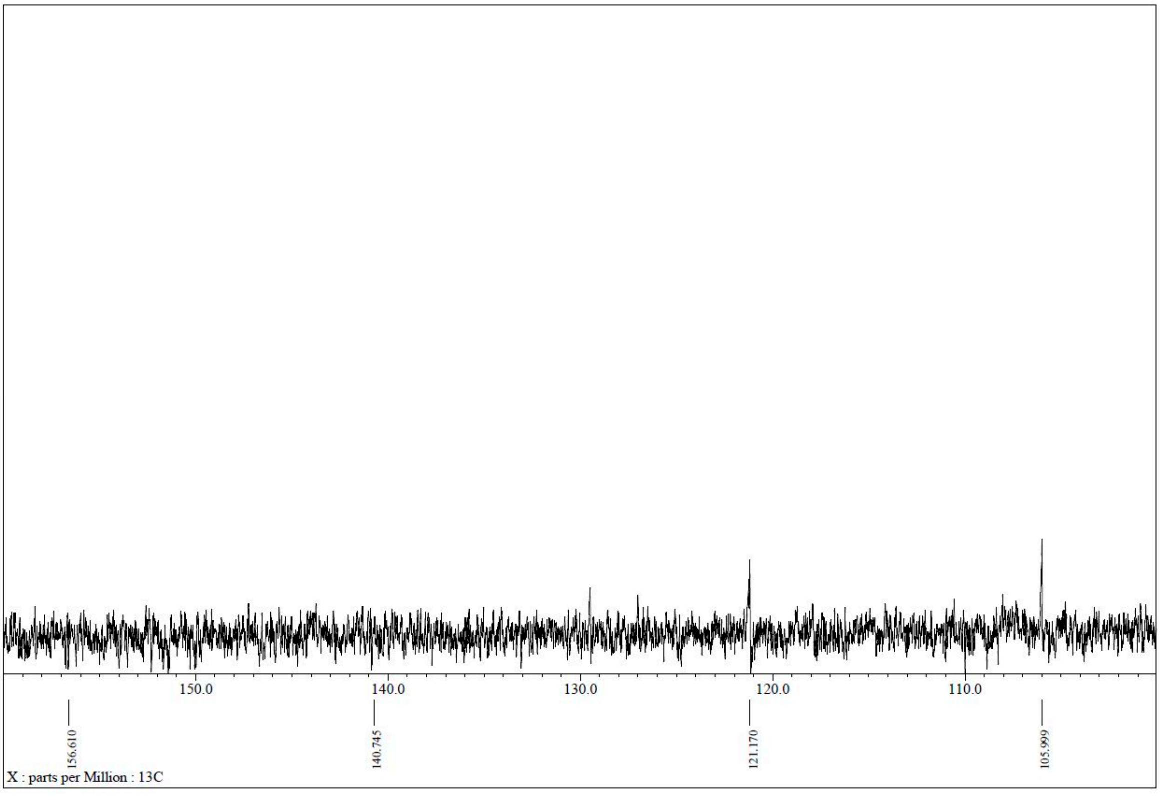

S20. ^{13}C NMR (100−160 ppm) spectrum (100 MHz) of compound **2** in $CDCl_3$.

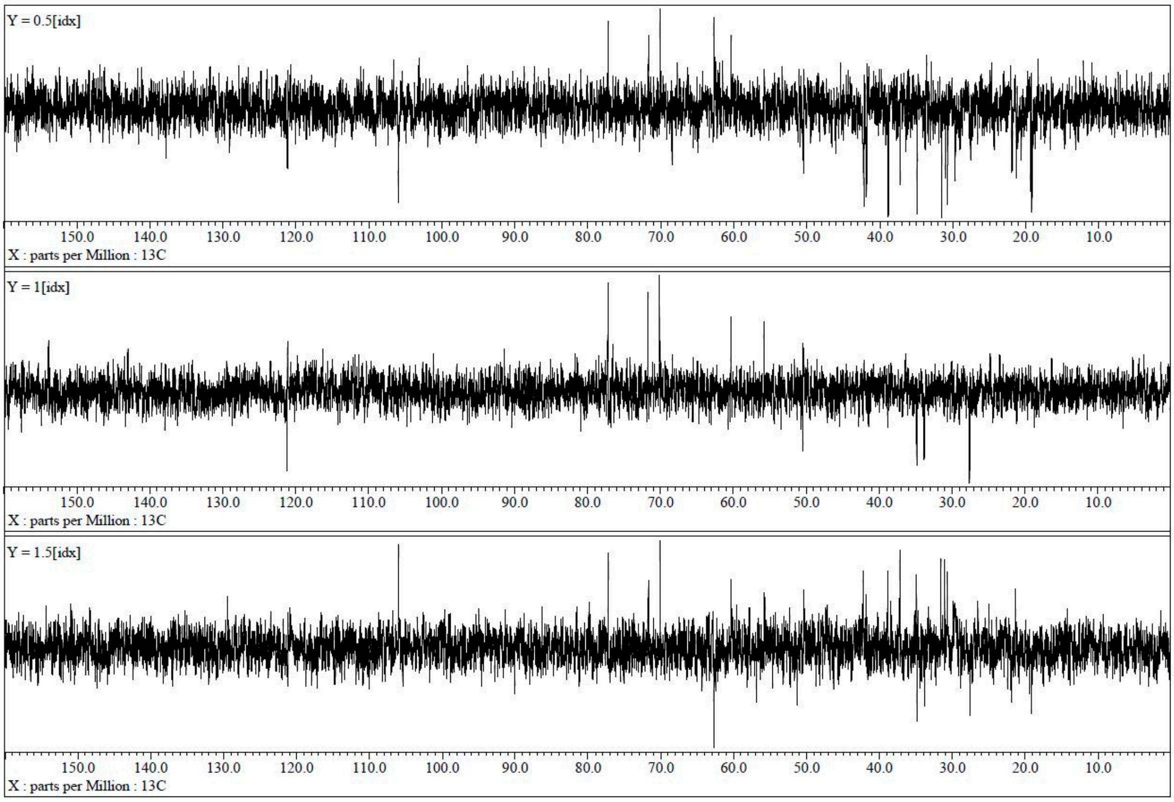

S21. DEPT spectrum of compound **2** in $CDCl_3$.

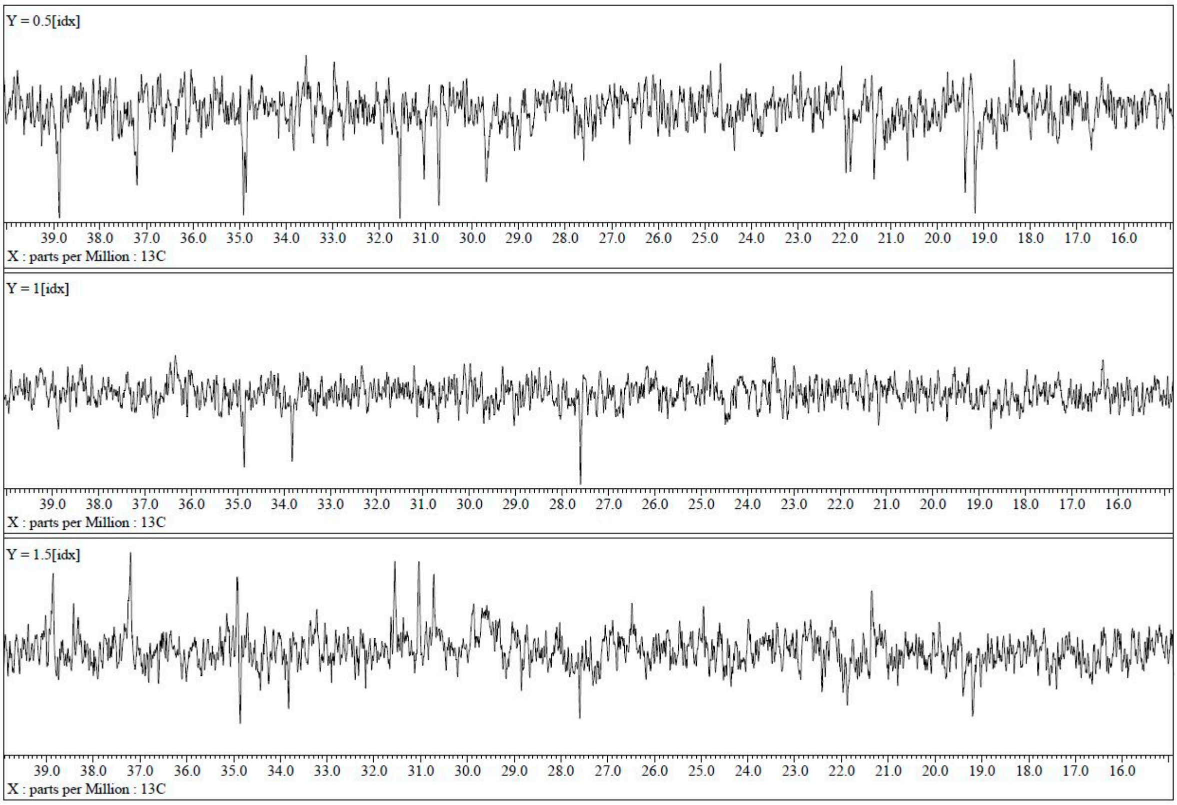

S22. DEPT (15–40 ppm) spectrum of compound **2** in $CDCl_3$.

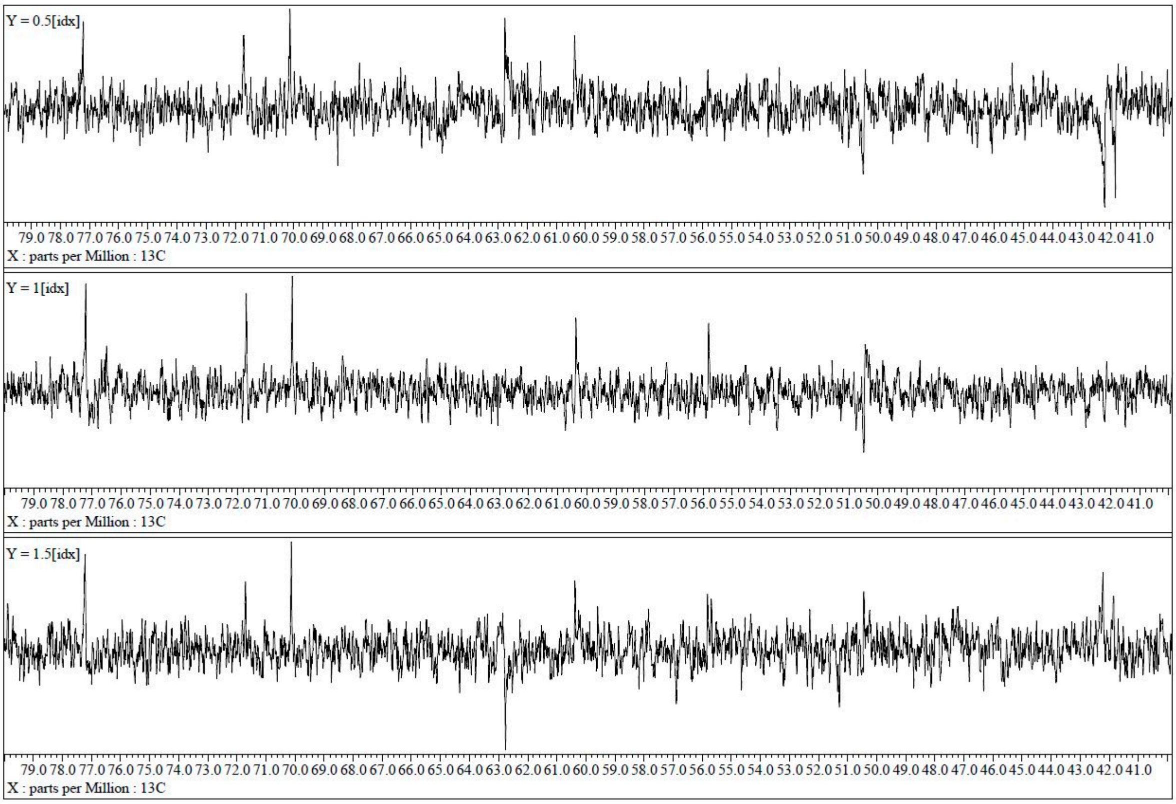

S23. DEPT (40−80 ppm) spectrum of compound **2** in $CDCl_3$.

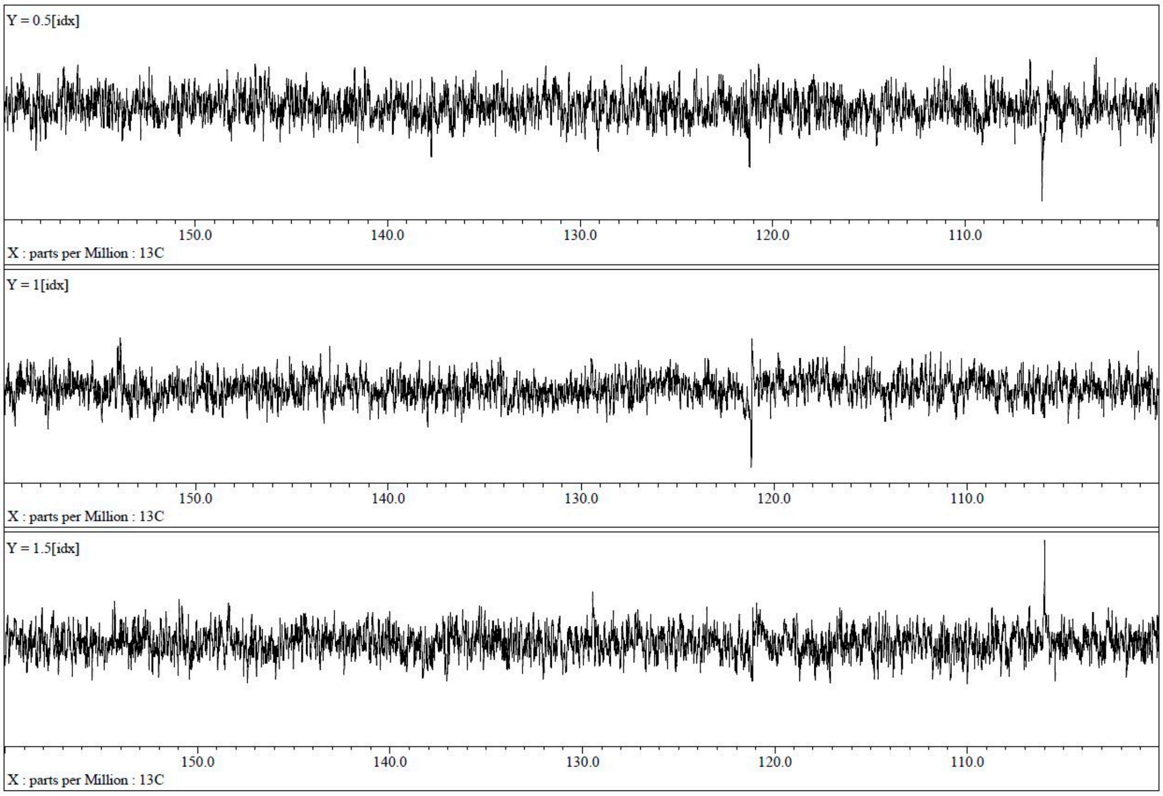

S24. DEPT (100−160 ppm) spectrum of compound **2** in $CDCl_3$.

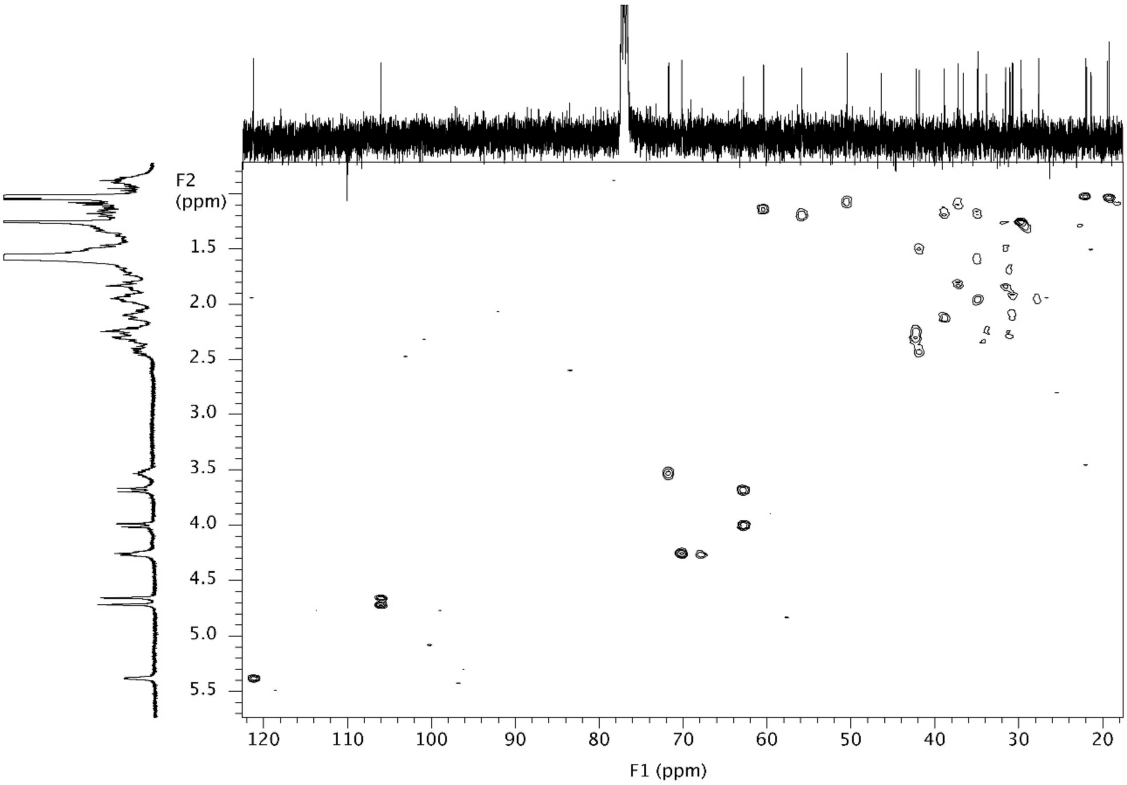

S25. gHSQC spectrum of compound **2** in $CDCl_3$.

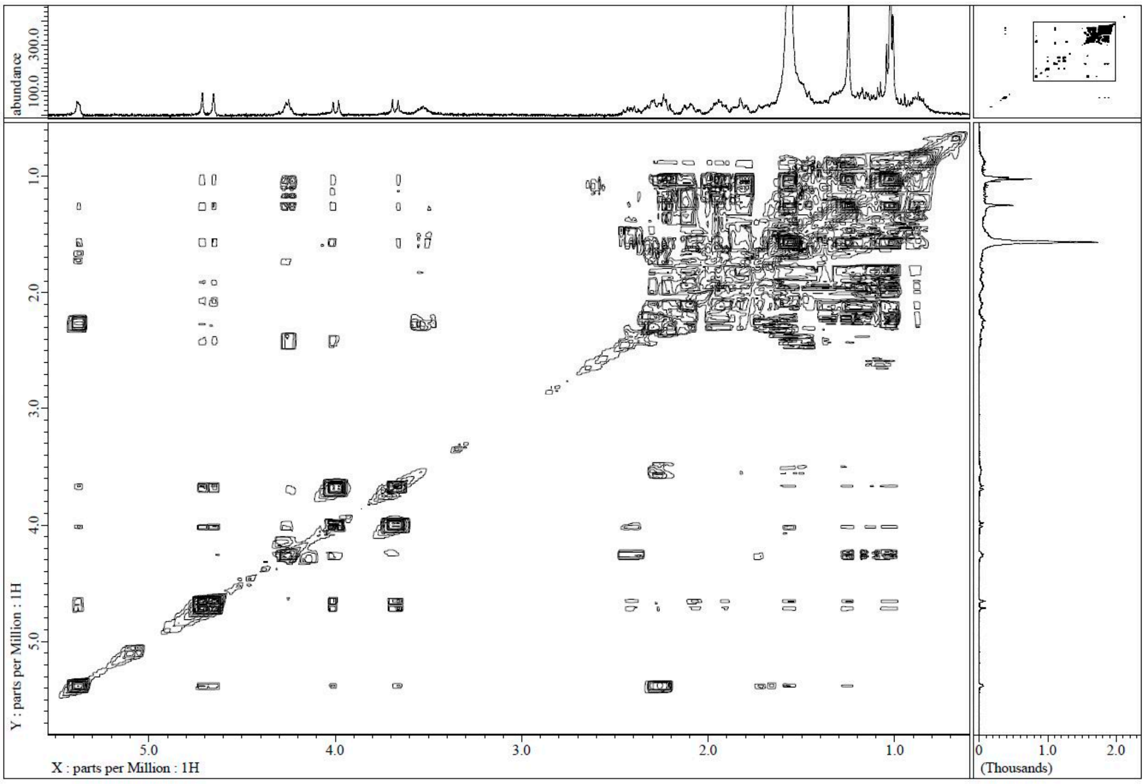

S26. ^{1}H–^{1}H COSY spectrum of compound **2** in $CDCl_3$.

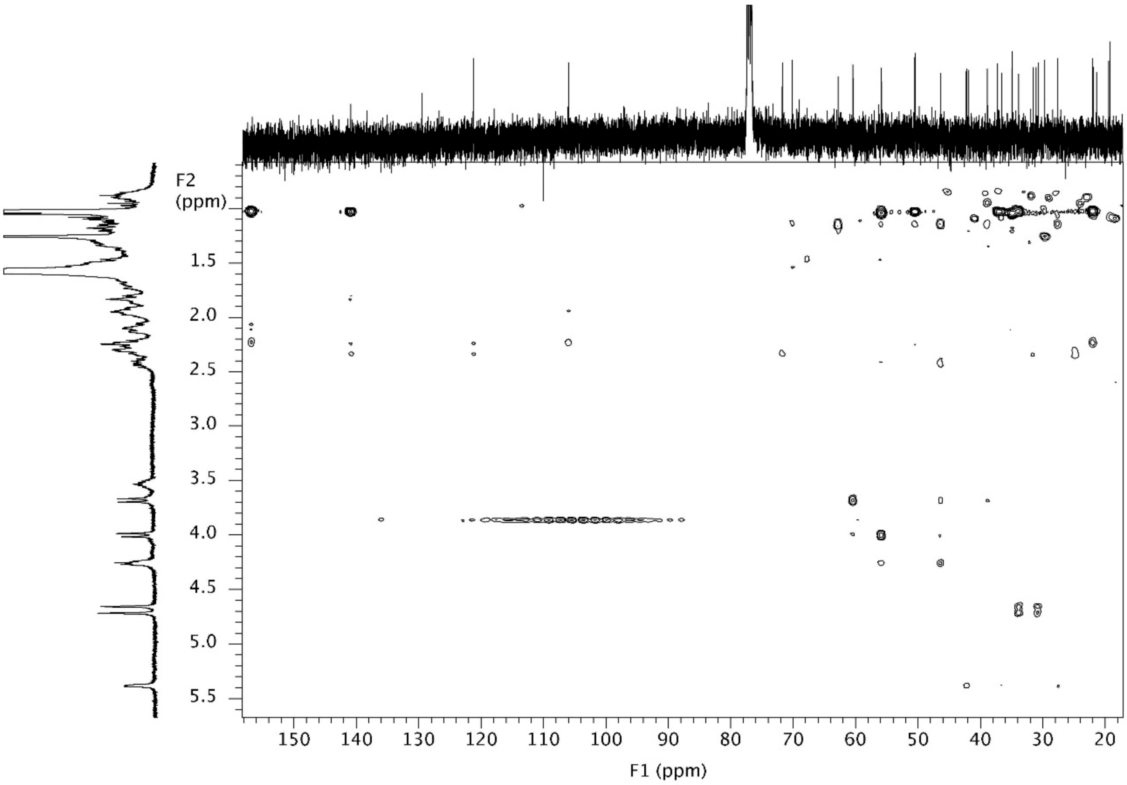

S27. gHMBC spectrum of compound **2** in $CDCl_3$.

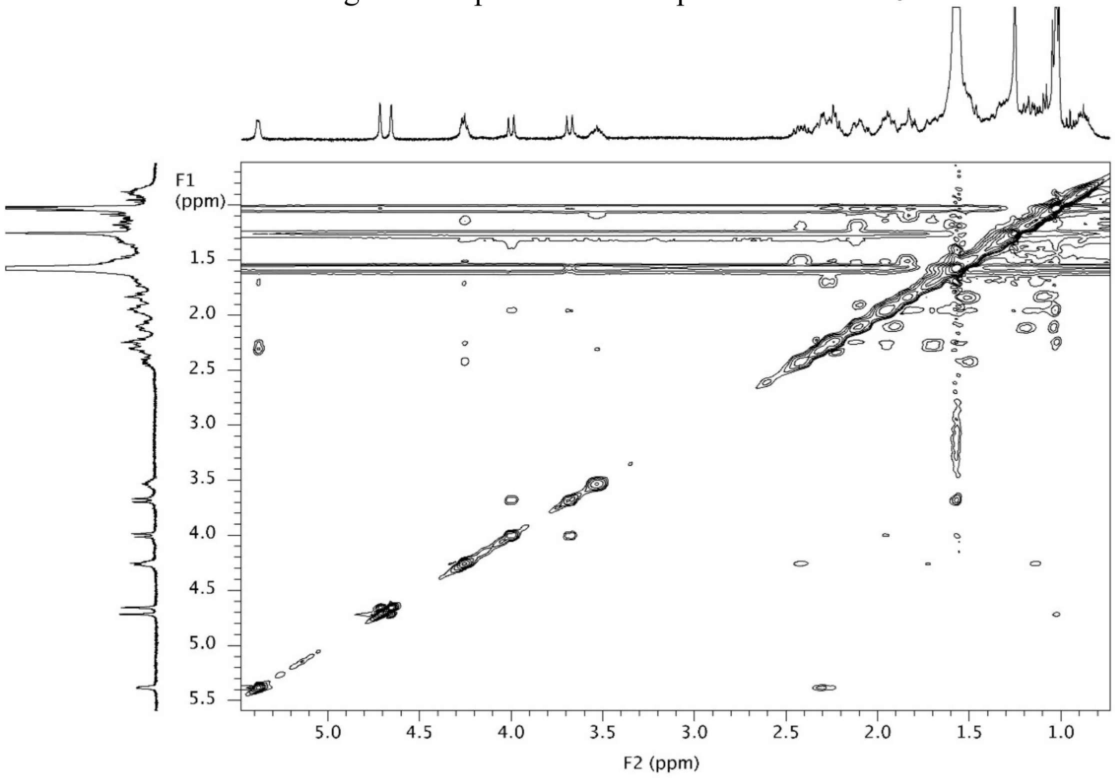

S28. NOESY spectrum of compound **2** in $CDCl_3$.
